# Adaptation to pitch-altered feedback is independent of one’s own voice pitch sensitivity

**DOI:** 10.1038/s41598-020-73932-1

**Published:** 2020-10-08

**Authors:** Razieh Alemi, Alexandre Lehmann, Mickael L. D. Deroche

**Affiliations:** 1 Department of Otolaryngology, Faculty of Medicine, McGill University, Montreal, QC Canada; 2 Centre for Research on Brain, Language and Music (CRBLM), Montreal, QC Canada; 3 International Laboratory for Brain, Music and Sound Research (BRAMS), Montreal, QC Canada; 4 Laboratory for Hearing and Cognition, Department of Psychology, Concordia University, Montreal, QC Canada

**Keywords:** Auditory system, Psychology

## Abstract

Monitoring voice pitch is a fine-tuned process in daily conversations as conveying accurately the linguistic and affective cues in a given utterance depends on the precise control of phonation and intonation. This monitoring is thought to depend on whether the error is treated as self-generated or externally-generated, resulting in either a correction or inflation of errors. The present study reports on two separate paradigms of adaptation to altered feedback to explore whether participants could behave in a more cohesive manner once the error is of comparable size perceptually. The vocal behavior of normal-hearing and fluent speakers was recorded in response to a personalized size of pitch shift versus a non-specific size, one semitone. The personalized size of shift was determined based on the just-noticeable difference in fundamental frequency (F0) of each participant’s voice. Here we show that both tasks successfully demonstrated opposing responses to a constant and predictable F0 perturbation (on from the production onset) but these effects barely carried over once the feedback was back to normal, depicting a pattern that bears some resemblance to compensatory responses. Experiencing a F0 shift that is perceived as self-generated (because it was precisely just-noticeable) is not enough to force speakers to behave more consistently and more homogeneously in an opposing manner. On the contrary, our results suggest that the type of the response as well as the magnitude of the response do not depend in any trivial way on the sensitivity of participants to their own voice pitch. Based on this finding, we speculate that error correction could possibly occur even with a bionic ear, typically even when F0 cues are too subtle for cochlear implant users to detect accurately.

## Introduction

Fluent speech in daily conversation is believed to be controlled by a sophisticated neural network, including the motor, somatosensory, and auditory systems^1^. Theoretical frameworks argue that the first communication attempts, e.g., babbling and speech-like productions, are the main sources for the motor system to develop the basic ready-to-perform speech commands. Step by step, these commands serve to predict the actions, reduce neural reaction time during articulation, and enhance speech fluency^2–7^.

By getting older and through different developmental stages, dramatic changes in the hormonal system and the structural shape and size of the speech articulators force speakers to precisely monitor and adjust their speech output to fulfill the communication goals^8–10^. From those moments on, somatosensory and acoustic feedback information play pivotal roles in recalibrating the stored motor commands and maintaining speech accuracy and fluency^1,2,11–15^. Some believe that these refined motor commands would last for a long time through adulthood, even when a speaker loses his sense of hearing^4,16–19^.

Altered auditory feedback is a well-known paradigm employed to investigate the role of auditory feedback in monitoring speech production. In this paradigm, a change is induced in the feedback while a person is speaking, thereby generating an error between the expectation of the vocal sound based on motor commands and the perceived auditory input. The expected behavior from the speaker is to detect the error and correct it^2^. Feedback manipulation could target different acoustical parameters including fundamental frequency of the voice (F0)^3–5,17,19–22^, formant frequencies of vowels^6,23–25^, and fricative consonants^26^. As an example, speakers whose voice is manipulated downwards in F0 will tend to shift their vocal range slightly upwards, as a way to compensate for the error perceived. These compensatory responses are often independent of the direction of the F0 shift^3,15,27,28^. In addition, experimental paradigms are divided based on their timescale. The induced perturbation could be either sudden and random within a trial, or predictable and constant across multiple trials. In the former type, the focus of analyses is on immediate within-trial compensatory responses to the altered feedback. In the latter type, as in the current study, the focus is on subjects progressively updating internal commands specific to the laryngeal control (e.g., to monitor voice F0) or specific to the movements of vocal tract articulators (e.g., to monitor formants). Since the auditory feedback manipulation is predictable and constant across trials, speakers gradually show long-term changes in the internal motor commands. This adaptive behavior that happens due to motor map recalibration is called *sensorimotor adaptation* and is believed to be a strategy recruited by the motor system to prevent future mismatches^4,5,23,29,30^.

Interestingly, studies using F0 manipulation paradigms showed that not all speakers compensate for voice pitch manipulation. Some actually follow the direction of the shift with similar or smaller response magnitude than the compensatory responses in both predictable^3^ and unpredictable^31–34^ settings. The exact reason why speakers would oppose or follow errors has been a continuous matter of debate. One speculation is that speakers might exhibit following responses when they compare the heard pitch with an external reference, e.g., singing in a choir group and realizing that their voice is slightly off from the group. In contrast, they would exhibit opposing responses when detecting a mismatch with an internal reference, i.e., memory system or the perceived feedback^3^. This problem is not trivial since defining a reference point does not guarantee consistency in vocal behavior because the same speaker might exhibit both types of responses across trials within the same experiment^29,34^. Therefore, one can reasonably remain dubious about the logic behind these two opposite behaviors in response to altered auditory feedback. Further investigation is warranted, especially when considering that adaptation to altered feedback might be outside of conscious control^15,17^.

The size of the pitch alteration might be another important factor affecting the type of responses. Some suggested that small shifts lead to opposing behavior, whereas large shifts result in following responses^3^ . Computational models corroborated this idea to some degree by predicting that the brain should ignore large errors in the auditory feedback as being an instance of self-generated speech^35^. This theoretical prediction was subsequently confirmed by a recent study. Taking advantage of a compensation paradigm, Korzyukov et al.^36^ showed that the “Sense of Agency” toward the auditory feedback, i.e., a sense of being the creator of the heard sound, helped speakers trigger an error correction process. As the pitch manipulation exceeded 250–300 cents, speakers disregarded the auditory feedback as being their own (self-generated) vocalization and the magnitude of their vocal responses toward the induced errors decreased. Put it differently, it is reasonable to infer that the brain would operate in an opposing manner provided that it considers the perceived pitch as a self-generated production and in a following manner when it considers the auditory feedback as an alien one. If this interpretation were correct, it would mean that the brain should possess a specific criterion to judge the “familiarity” or “self-identity” of a given vocal production, and this preliminary identification would be a critical factor in generating a correction or inflation of the error. Tian and Poeppel^37^ referred to this criterion as “spectral integration window”. Using two behavioural experiments and a mental imagery paradigm, the authors showed that speakers consider the auditory feedback as their own production provided that the internal prediction and external feedback remains temporally and spectrally close to each other.

However, these criteria fail to represent the individual differences reported by different studies. It is possible that some of the variability observed across participants in compensation or adaptation paradigms could originate from the different F0 sensitivity that these participants possessed. Some listeners with a fine sense of pitch (e.g. a just noticeable differences (JND) of 20 cents) could have considered a 100-cent shift as externally-generated, whereas other listeners with a poor sense of pitch (e.g. a JND of 150 cents) would have treated the same perturbation as self-generated. In other words, in order to better understand why behaviors may be just as expected or very erratic in altered-feedback paradigms, it may be necessary to measure each participant’s sensitivity to the error, translating here into each participant’s sense of their own voice pitch.

There is a relatively broad range of F0 JNDs across speakers, and the idea that this could participate to the inter-speakers’ variability in response to F0 perturbation is not new, as speakers with high discriminative auditory acuity are more likely to notice errors in their speech production^11,12,38^. Inspired by this suggestion, Villacorta et al.^39^ investigated the influence of auditory acuity over the degree to which speakers compensated for the perturbations of the first formant frequency of vowels. They reported a significant correlation between these two measures. With a similar hypothesis and in an attempt to draw a finer picture of the relationship between auditory acuity and adaptation to formant-shifted feedback, Martin et al.^30^ took advantage of a series of auditory discrimination tasks and reported that adaptation to manipulated feedback was predicted by assessing pitch, loudness, melody and transposed melody discrimination scores, which they referred to as general auditory acuity. Their results confirmed previous findings implying that speakers with poor auditory acuity are less competent in detecting and correcting feedback errors. Recently, Murray and Stepp^22^ examined the relationship between auditory discrimination and vowel production. They asked both adults and children to perform three tasks, including a two-alternative forced-choice pitch discrimination task, a compensation, and an adaptation paradigm. The authors reported a significant effect of JND on the amount of adaptation to pitch-shifted auditory feedback, meaning that participants with higher level of pitch discrimination ability showed greater adaptation. However, the authors failed to observe this effect of pitch sensitivity on the compensatory responses.

On the other hand, evidence for a lack of relationship has been reported by Feng et al.^40^ for the correlation between adaptation to F1 manipulation and auditory acuity for F1, Cai et al.^41^ for the correlation between speaker’s JNDs toward the F1 manipulation and compensatory responses to F1 manipulations, Abur et al.^42^ for the correlation between adaptation to F0 manipulation and F0 JND, and most recently, Smith et al.^43^ for the correlation between compensatory responses to F0 manipulation and F0 JND.

In the present study, we pushed this research topic further by questioning whether this link might be observed when acuity is measured directly on the participants’ voice. After all, what is the point of having a very fine sense of pitch for violins and trumpets if the goal is to control one’s own speech? The relevant acuity for such investigations is one that must be personal. Thus, we devised a protocol that aimed to measure auditory acuity to one’s own voice pitch and compare it to the magnitude of the adaptation response to a fixed (100 cents) alteration, or an alteration of the JND size. Moreover, instead of relying on the traditional method, we developed a new analysis to measure the magnitude of adaptation from the intact vocalizations produced at the beginning and the end of the paradigm. The reason behind this analysis optimization was the upward or downward trends in F0 production commonly observed across entire experiments. Such trends often interfere with the assessment of opposing or following behavior and make the categorization/labeling inaccurate when it is based uniquely from vocalizations at the beginning of the paradigm. This problem can be easily overcome by taking advantage of the very last productions (once feedback is back to normal, i.e. “washout phase”), provided that any after-effect has dropped (an assumption we examined very carefully).

We addressed two major hypotheses. First, we hypothesized that a great deal of variability in the sensorimotor adaptation to a fixed 100-cent shift is due to the differential F0 sensitivity of different subjects, i.e., subjects not correcting to the same degree because they do not detect errors with the same accuracy, regardless of the direction of F0 manipulation. As a consequence, we expected to observe (1) a more homogeneous adaptation magnitude across subjects in response to the JND shift, and (2) a negative correlation between F0 JND and adaptation magnitude (similar to Murray and Stepp^22^). Second, adjusting the size of the F0 alteration to the participant’s JND should, in principle, optimize participants’ belief that they hear their own voice and not an alien or externally-generated voice. If familiarity/self-identity is the key, then we should observe larger adaptation magnitudes when the F0 shift is personalized.

Finally, the practical need to measure adaptation twice—one before and one after F0-JND estimation – led to additional insight. For example, we could examine whether participants were consistent in the nature of their responses, akin to a test/retest reliability exercise, leading to a close look at the conditions that caused following responses. Also, both F0 JND and adaptation magnitude could, in principle, depend on the vowel being produced (because spectral envelope cues might play a role in F0 coding, or vocal cords might be easier to control with a large opening of the mouth). Therefore, we examined whether these outcome measures varied significantly between three vowels, or whether they could be more consistent between test/retest with one vowel over another.

Finally, one last reason behind designing this study was our clinical interest in examining, in the future, the vocal behavior of cochlear implant users in response to F0 manipulation. The auditory feedback in people with cochlear implants is constant and predictable, but far from precise and “tuned”. Therefore, the adaptation paradigm is uniquely relevant (in a way that compensation paradigms are not) as pitch errors are likely common but do not arise unexpectedly in these devices.

## Results

The results of the current study are presented in four sections. The first section discusses the results of the first experiment, where the size of the pitch shift was 100 cents for all participants. The second section describes the F0 JND values estimated from the participants in response to their own vocalizations. The third section presents the results of the third experiment, i.e. the adaptation paradigm with a personalized size of the F0 shift. Finally, the relationships between the amount of adaptation taken from the first and third experiments and some relevant factors are explored in the fourth section. Results are illustrated in the figures as means ± standard errors (SE) and written in the following sections as means ± standard deviations (STD). Mauchly’s tests of sphericity were performed for each analysis, and Greenhouse–Geisser correction was applied whenever needed.

### First experiment

#### Adaptation to 100 cents F0-shift

Even though participants were randomly assigned to the two groups (assigned to either upward or downward shift in F0), we ran a preliminary check to verify that the two groups did not differ in any respect to begin with. The mean age of participants who experienced downward and upward shift directions were 32.6 (± 11.2) and 33.6 (± 9.1) years, respectively, with no significant difference between them [t (57) = 0.395, *p* = 0.694]. The mean of F0 (in Hz) for the baseline trials were 167.7 ± 49.2 Hz (16 Female, 14 male) in the group assigned to the downward pitch shift and 178.0 ± 45.8 Hz (16 Female, 13 Male) in the group assigned to upward pitch shift, with no significant difference between them [t (57) = 0.8, *p* = 0.397 for mean F0; t (57) = 1.1, *p* = 0.257 for F0 STD].

**Figure 1:**
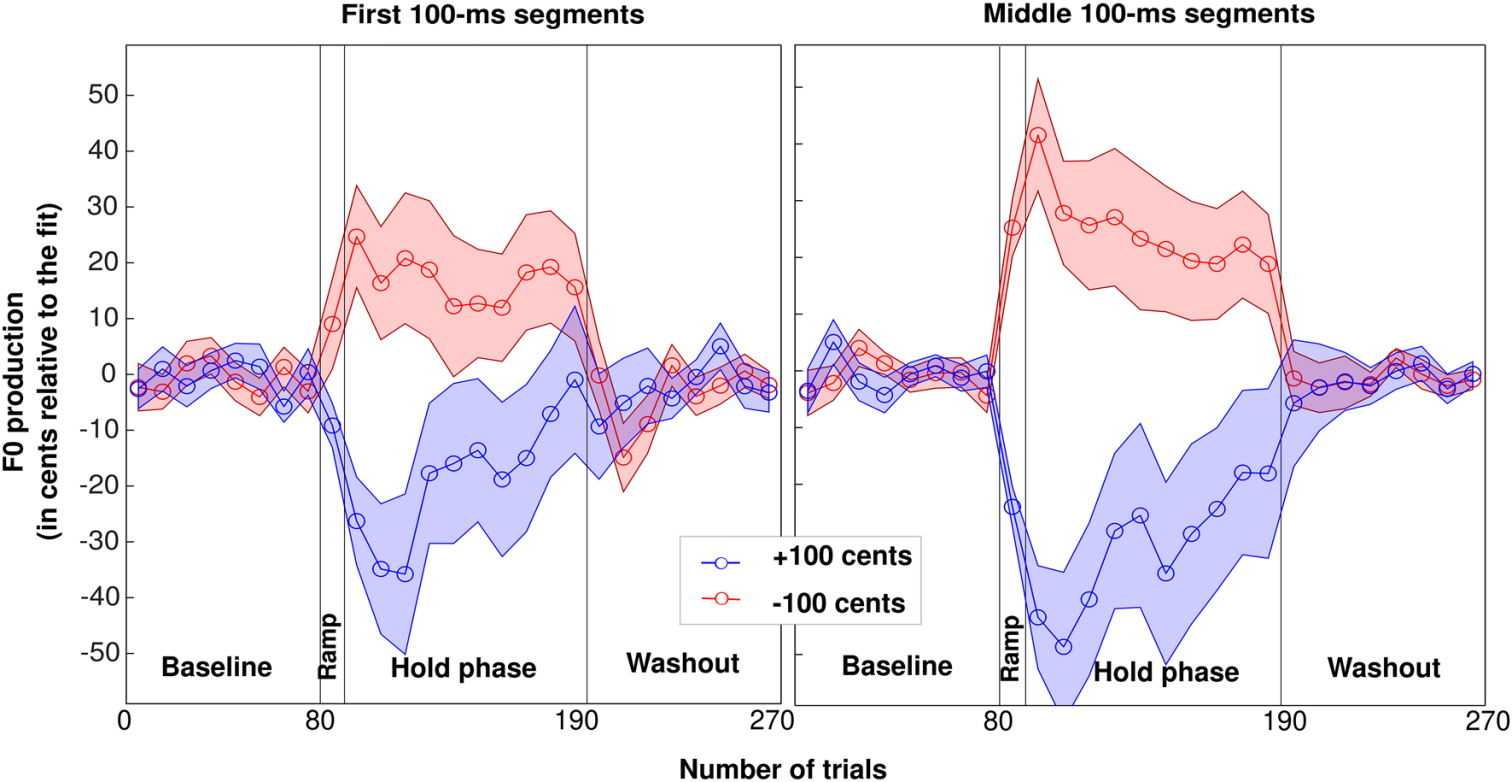
Group-level average of the vocal performance of participants who experienced ± 100 cents F0 shift, depicted within the first 100 ms (left) and the middle 100 ms (right) portions of vocalizations. In this plot, the vertical scale indicates how much adaptation participants exhibited, on average, in response to the perceived errors. The red and blue lines (± SE) represents the vocal behavior in the downward and upward shift directions, respectively. SE: standard error.

More relevant to the goal of this study, Fig. 1 depicts the group-level average of vocal behavior of participants who experienced ± 100 cents F0-shift, when analyses focussed on the first 100-ms segments (left panel) or the middle 100-ms segments (right panel). Qualitatively, there were three main results: (1) there was overall an opposing response to the pitch shift, regardless of its direction, (2) this opposing behavior tended to fade away over the 100 trials of the hold phase, despite the size of the shift being constant at 100 cents, and 3) there was no evidence of any after-effect. To support these statements, a mixed repeated-measures ANOVA with one between-subject factor (shift direction) and two within-subject factors (vowel type and trial window, i.e. first/second half of the hold phase) was performed on the normalized F0 values (in cents, relative to the polynomial fit, and flipped for the upward pitch-shift group—blue symbols—for a fair comparison between the two groups). This was done separately for the first or middle segments. A similar ANOVA was performed with only vowel as a factor, for the first three blocks of the washout phase.

First 100-ms: the sphericity assumption was met for the main effect of vowels [Chi2(2) = 3.3, *p* = 0.196], and its interaction with trial window [Chi2(2) = 5.9, *p* = 0.052]. There was no main effect of group [F(1,57) < 0.1, *p* = 0.889], no effect of vowels [F(2,114) = 0.9, *p* = 0.423] or first/second half of the hold phase trials [F(1,57) = 3.3, *p* = 0.076], and any 2- or 3-way interaction missed significance [*p* > 0.215]. Ignoring vowel, a simple t-test revealed a significant opposing response over this hold phase [t(58) = 2.6, *p* = 0.011; mean = 18.3 cents]. Further, we assessed the after-effect: there was no effect of group [F(1,57) = 2.8, *p* = 0 0.099], no effect of vowel [F(2,114) = 1.2, *p* = 0.305] but a significant interaction [F(2,114) = 7.5, *p* = 0.001]. This interaction was not particularly interesting since the after-effect could not reach significance in any of them; ignoring vowel, it was not significantly different from 0 [t(58) = − 0.3, *p* = 0.756]. This lack of after-effect was unexpected, and thus we wondered whether participants could have adapted back to the normal feedback so quickly that it would only be observable in the first few trials. As we forced at least 3 occurrences of each vowel per block of 10 trials, we could examine no fewer than 8 trials to include vowel as a factor, but we also looked at 5 trials only, ignoring vowel. With the first 8 trials of the washout phase: there was no effect of group [F(1,57) = 1.4, *p* = 0.251], no effect of vowel [F(2,114) = 0.4, *p* = 0.658] and no interaction [F(2,114) = 1.7, *p* = 0.187]. A simple t-test did not reveal any after-effect within these 8 trials [t(58) = 1.1, *p* = 0.288] or even the first 5 trials [t(58) = 1.4, *p* = 0.166].

Middle 100-ms: the sphericity assumption was met for the effect of vowels [Chi2(2) = 2.9, *p* = 0.235], but it was violated for the interaction between vowel type and trial window [Chi2(2) = 27.8, *p* < 0.001]. Greenhouse–Geisser correction was used to adjust its degree of freedom. There was no main effect of group [F(1,57) = 0.1, *p* = 0.721], but a main effect of vowels [F(2,114) = 6.3, *p* = 0.003] and first/second half of the hold phase trials [F(1,57) = 4.0, *p* = 0.050], while any 2- or 3-way interactions missed significance [*p* > 0.109]. Ignoring vowel, there was a significant opposing response in the first half of the hold phase [t(58) = 4.2, *p* < 0.001; mean = 33.7 cents] and the second half to a smaller degree [t(58) = 2.7, *p* = 0.008; mean = 23.4 cents]. Ignoring first/second half, the opposing response was significant with each vowel [t(58) = 4.5, 3.0, and 3.0; *p* < 0.001, *p* = 0.004, and *p* = 0.004, for /a/, /e/, and /o/, respectively], being slightly stronger with /a/ (36.5 cents) than with /e/ (24.8 cents) or /o/ (24.4 cents). Further, we assessed the after-effect: there was no effect of group [F(1,57) = 0.3, *p* = 0.584], no effect of vowel [F(2,114) = 0.5, *p* = 0.607] but a significant interaction [F(2,114) = 4.3, *p* = 0.015]. Once again, this interaction was not particularly meaningful given that the after-effect was not significant in any vowel; ignoring vowel, it was not significantly different from 0 [t(58) = 0.2, *p* = 0.859].

Here again, we wondered whether participants could have exhibited an after-effect but only within the first few trials. So, within the first 8 trials of the washout phase: there was no effect of group [F(1,57) = 0.5, *p* = 0.507], no effect of vowel [F(2,114) = 0.2, *p* = 0.801] and no interaction [F(2,114) = 0.8, *p* = 0.452]. A simple t-test did not reveal any after-effect within these 8 trials [t(58) = 0.2, *p* = 0.800] or even the first 5 trials [t(58) = 0.5, *p* = 0.641].

To summarize, the first experiment successfully demonstrated an opposing response to this adaptation paradigm that disregarded the direction of the 100-cent shift, but importantly, this response did not carry over once the feedback was back to normal. Results were largely similar whether analyses focussed on the first 100-ms or the middle 100-ms, the main differences being: (1) stronger responses in the middle rather than the onset of productions, and (2) stronger responses with vowel /a/ than vowels /e/ and /o/, in the middle of the productions, and (3) the opposing behavior to the constant perturbation tended to decrease in magnitude over time and this was easier to observe looking at the middle rather than the onset of productions.

### Second experiment

#### F0 JNDs

**Figure 2:**
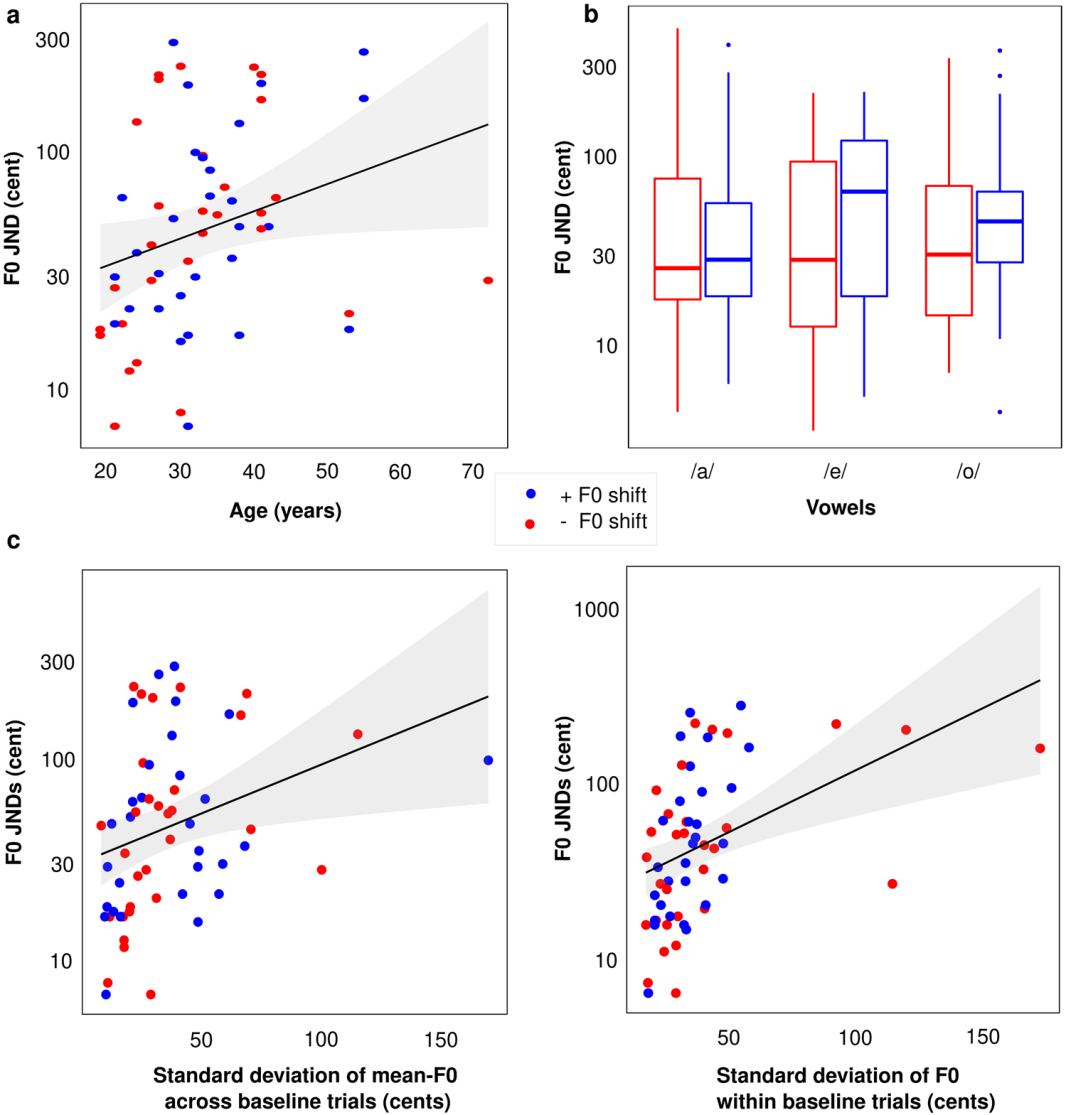
(**a**) F0 JNDs across participants as a function of their age. (**b**) The mean and distribution of F0 JNDS specific for each vowel and each group are depicted in boxplots. (**c**) Correlations between F0 JNDs and across-trial (left) and within-trial F0 (right).

Figure 2 displays the averaged JNDs as a function of vowel and group. The F0 JNDs ranged from 7 to 295 cents, with a total average of 47.4 cents and vowel-specific JNDs of 39.5, 43.3, and 43.2 cents for vowels /a/, /e/, /o/, respectively. Mixed ANOVA with one between-subject factor (shift direction experienced in the first experiment) and one within-subject factor (vowel) was performed on the dependent variable (JNDs). We noticed that on a linear scale (of cents), the sphericity assumption was not met, so we log-transformed the JND values to perform this analysis. The sphericity assumption was respected for the effect of vowels [Chi2(2) = 4.7, *p* = 0.097]. There was no effect of group (or shift direction) [F(1,57) = 0.7, *p* = 0.391], no effect of vowel [(F(2, 114) = 0.6, *p* = 0.553], and no interaction between them [ F(2, 114) = 1.1, *p* = 0.341]. To summarize, F0 JNDs were similar regardless of the vowel and the group considered (either those assigned to + and − changes in F0 in experiment 1) (see Fig. 2).

Due to the wide age range of our participants (19–72 years), we conducted correlation analyses to assess whether age could have negatively affected the F0 JNDs of our participants. When JNDs were expressed on a linear scale (of cents), the correlation with age missed significance (r2 = 0.05, *p* = 0.078), but it reached significance (r2 = 0.07, p = 0.038) when expressing JNDs on a log scale, as illustrated in Fig. 2. Thus, age could have deteriorated pitch sensitivity to a small degree in this study.

The observation of several adults with a F0 JND beyond one semitone was quite unexpected. To find the reason behind this wide range of JNDs, we noticed that some recordings (on which this F0 discrimination task was built upon) were particularly variable in their F0 contour, so we examined the stability of F0 of each participant within baseline trials of the first experiment. We found that both the standard deviation of F0 within a trial (averaged over the 80 trials) and the standard deviation of the mean F0 (averaged over time in each trial) across the 80 trials were positively correlated with F0 JNDs (r2 = 0.19, *p* = 0.001 and r2 = 0.10, *p* = 0.016, respectively) (see bottom panels of Fig. 2). These results confirmed that variability inherent to the recordings generated some distraction that elevated JNDs in some participants. This is presumably why 14 participants had JNDs above 100 cents.

### Third experiment

#### Personalized adaptation paradigm

**Figure 3:**
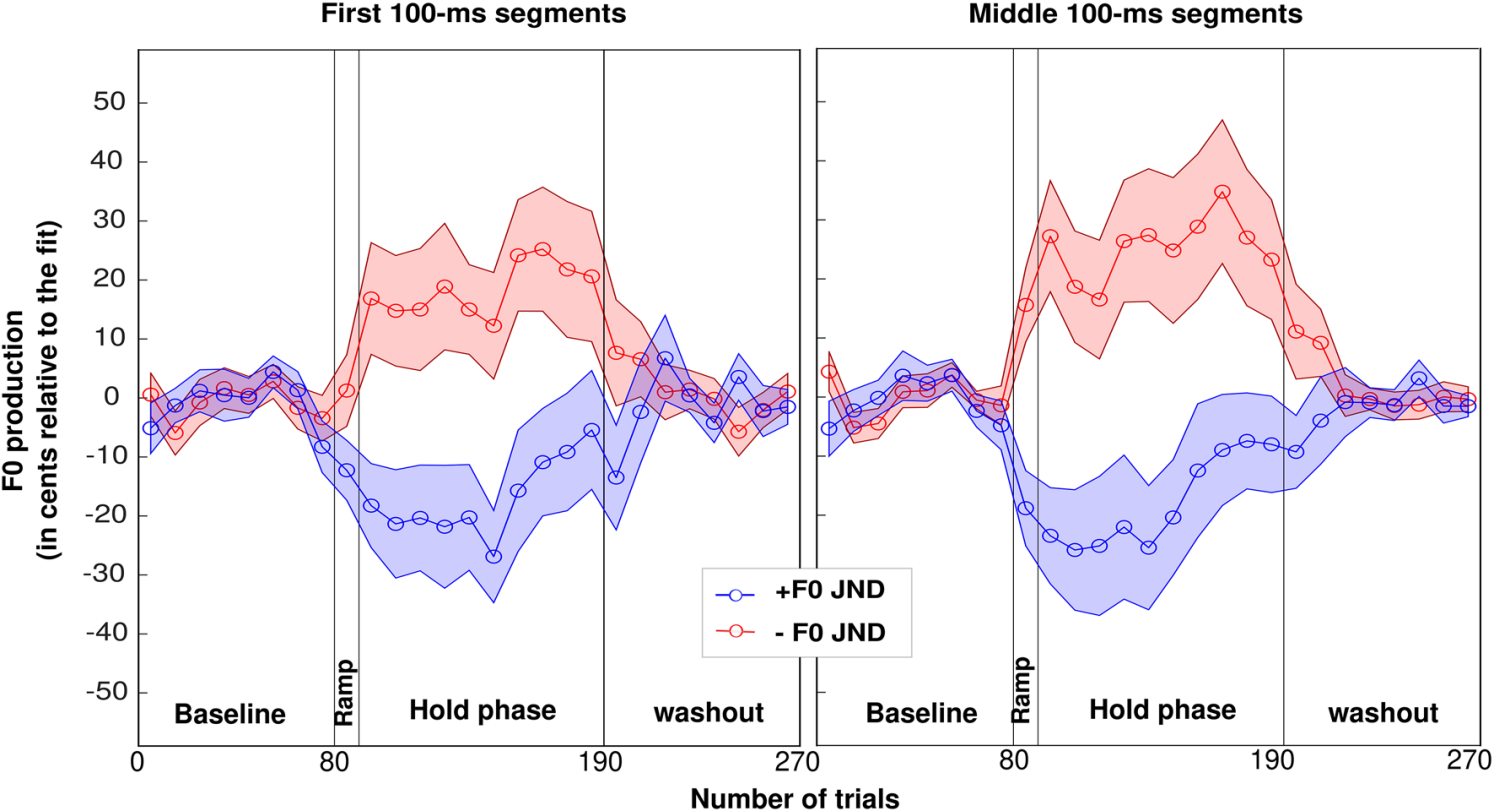
Group-level average of vocal performance of participants who experienced an F0 shift equal to their F0 JNDs, depicted within the first 100 ms (left) and the middle 100 ms (right) portions of vocalizations. The red and blue lines (± SE) represents the vocal behavior in response to downward and upward shift directions in the auditory feedback. SE: standard error.

Similar to the first experiment, we ran a preliminary check to verify that the two groups did not differ in any respect that would be irrelevant to the experimental manipulation. The mean of vocal F0s for the baseline trials in participants experiencing downward or upward shift directions were 163.9 ± 49.4 Hz and 176 ± 43.3 Hz, respectively, with no significant difference between them [t(57) = 1.0, *p* = 0.321 for mean F0; t(57) > 0.1, *p* = 0.915 for F0 STD]. More relevant to the goal of the third experiment, Fig. 3 depicts the group-level average of vocal behavior of participants who experienced an F0 shift equal to their F0 JNDs. Qualitatively, there were three main results: (1) there was overall an opposing response to the F0 shift, (2) the opposing response tended to fade away in one group but increased in the other as trials progress while the perturbation was maintained, and (3) there was no evidence of any after-effect. The same statistical approaches were conducted as in the first experiment.

First 100-ms: the sphericity assumption was violated for both the effect of vowels [Chi2(2) = 22.4, *p* < 0.001], and its interaction with trial window [Chi2(2) = 17.1, *p* < 0.001]. Greenhouse–Geisser corrections were used to adjust degrees of freedom. There was no main effect of group [F(1,57) < 0.1, *p* = 0.873], no effect of vowels [F(1.5,87.5) = 1.1, *p* = 0.326] or first/second half of the hold phase trials [F(1,57) < 0.1, *p* = 0.818], and any 2- or 3-way interactions missed significance [*p* > 0.183]. Ignoring vowel, a simple t-test revealed a significant opposing response over this hold phase [t(58) = 3.1, *p* = 0.003; mean = 15.2 cents]. Further, we assessed the after-effect. Again, the sphericity assumption was violated for vowels [Chi2(2) = 49.7, *p* < 0.001]. There was no effect of group [F(1,57) < 0.1, *p* = 0.764], no effect of vowel [F(1.3,71.8) = 1.6, *p* = 0.205], and no interaction [F(1.3,71.8) = 0.3, *p* = 0.632]. Ignoring vowel, a simple t-test failed to reveal any after-effect [t(58) = 0.9, *p* = 0.369]. As in the first experiment, we wondered whether the after-effect could have been short-lived within the first few trials before participants adapted quickly back to the normal feedback. There was no effect of group [F(1,57) < 0.1, *p* = 0.794], no effect of vowel [F(2,114) = 1.2, *p* = 0.307] and no interaction [F(2,114) = 0.8, *p* = 0.424] when including the first 8 trials of the washout phase. Yet, a simple t-test did reveal a significant after-effect within these 8 trials [t(58) = 2.1, *p* = 0.045], which was even stronger in the first 5 trials [t(58) = 2.2, *p* = 0.031].

Middle 100-ms: the sphericity assumption was violated for both the effect of vowels [Chi2(2) = 25.4, *p* < 0.001], and its interaction with trial window [Chi2(2) = 20.1, *p* < 0.001]. There was no main effect of group [F(1,57) = 0.3, *p* = 0.305], no effect of vowels [F(1.5,83.5) = 1.9, *p* = 0.170] or first/second half of the hold phase trials [F(1,57) = 1.5, *p* = 0.229]. There was a 2-way interaction between group and trial window [F(1,57) = 5.9, *p* = 0.018], caused by the opposing response fading away between the first to the second half of the hold phase in the group assigned to the upward shifts [F(1,57) = 6.5, *p* = 0.013], while the opposing response remained stable in the group assigned to downward shifts [F(1,57) = 0.8, *p* = 0.390]. No other interaction reached significance [*p* > 0.327]. Ignoring vowel, simple t-tests revealed a significant opposing response for the group assigned to downward shifts in the first [t(29) = 2.4, *p* = 0.023; mean = 22.9 cents] and second half [t(29) = 2.5, *p* = 0.020; mean = 27.4 cents] and only in the first half of the hold phase in the group assigned to downward shifts [t(28) = 2.4, *p* = 0.021; mean = 24.3 cents] but not in the second half [t(28) = 1.2, *p* = 0.231; mean = 11.0 cents]. Further, we assessed the after-effect. The sphericity assumption was violated for vowels [Chi2(2) = 66.4, *p* < 0.001]. Greenhouse–Geisser corrections were used to adjust degrees of freedom. There was no effect of group [F(1,57) < 0.1, *p* = 0.772], no effect of vowel [F(1.2,67.3) = 2.3, *p* = 0.129], and no interaction [F(1.2,67.3) = 0.3, *p* = 0.614]. Ignoring vowel, a simple t-test failed to reveal any after-effect [t(58) = 1.4, *p* = 0.166]. Looking closer at the first 8 trials of the washout phase, there was no effect of group [F(1,57) < 0.1, *p* = 0.802], no effect of vowel [F(2,114) = 2.2, *p* = 0.131] and no interaction [F(2,114) = 0.7, *p* = 0.446]. Yet, a simple t-test did reveal an after-effect within these 8 trials [t(58) = 1.9, *p* = 0.033] (or a borderline effect in the first 5 trials [t(58) = 2.2, *p* = 0.052]).

To summarize, the third experiment successfully demonstrated an opposing response to this individualized paradigm, but the response carried over only very briefly once the feedback was back to normal. Results were roughly similar whether analyses focussed on the first 100-ms or the middle 100-ms, the main difference being a stronger opposing response when analyses focussed on the middle of the productions rather than on their onset. Also, the opposing behavior to the constant perturbation tended to decrease in magnitude over time when analyzing the middle of productions, but it affected uniquely the group assigned to upward F0 shifts. As a reminder, F0 JNDs did not differ between the two groups (*p* = 0.601), and therefore both groups performed the adaptation paradigm in this session with comparable amounts of error induced. In other words, the size of the alteration could not explain why participants in the upward group failed to sustain the adaptation effect after several blocks of trials.

### Evaluating the consistency of vocal behavior across the two experiments

To compare the two adaptation paradigms, we focussed on the early F0 metric, as this was theoretically not confounded by compensatory influence affecting the middle-F0 metric. Note, however, that both metrics were highly correlated (e.g. in the hold phase: r2 = 0.69, *p* < 0.001 in the first experiment and r2 = 0.47, *p* < 0.001 in the third experiment). The average amount of adaptation in the first experiment was 17.8 cents (on average across all vowels, blocks, and participants) for an error fixed at 100 cents. In the personalized paradigm, the error induced was about half of what it was in the first experiment and yet, the adaptation magnitude was almost the same, 17.7 cents (on average across all vowels, blocks, and participants). However, one should bear in mind that the amount of adaptation or compensation is generally not proportional to the size of the error induced. Errors smaller than one semitone tend to generate larger effect size as a percentage of the shift^32^. So, this may well be why our individualized paradigm appeared quite effective.

**Figure 4:**
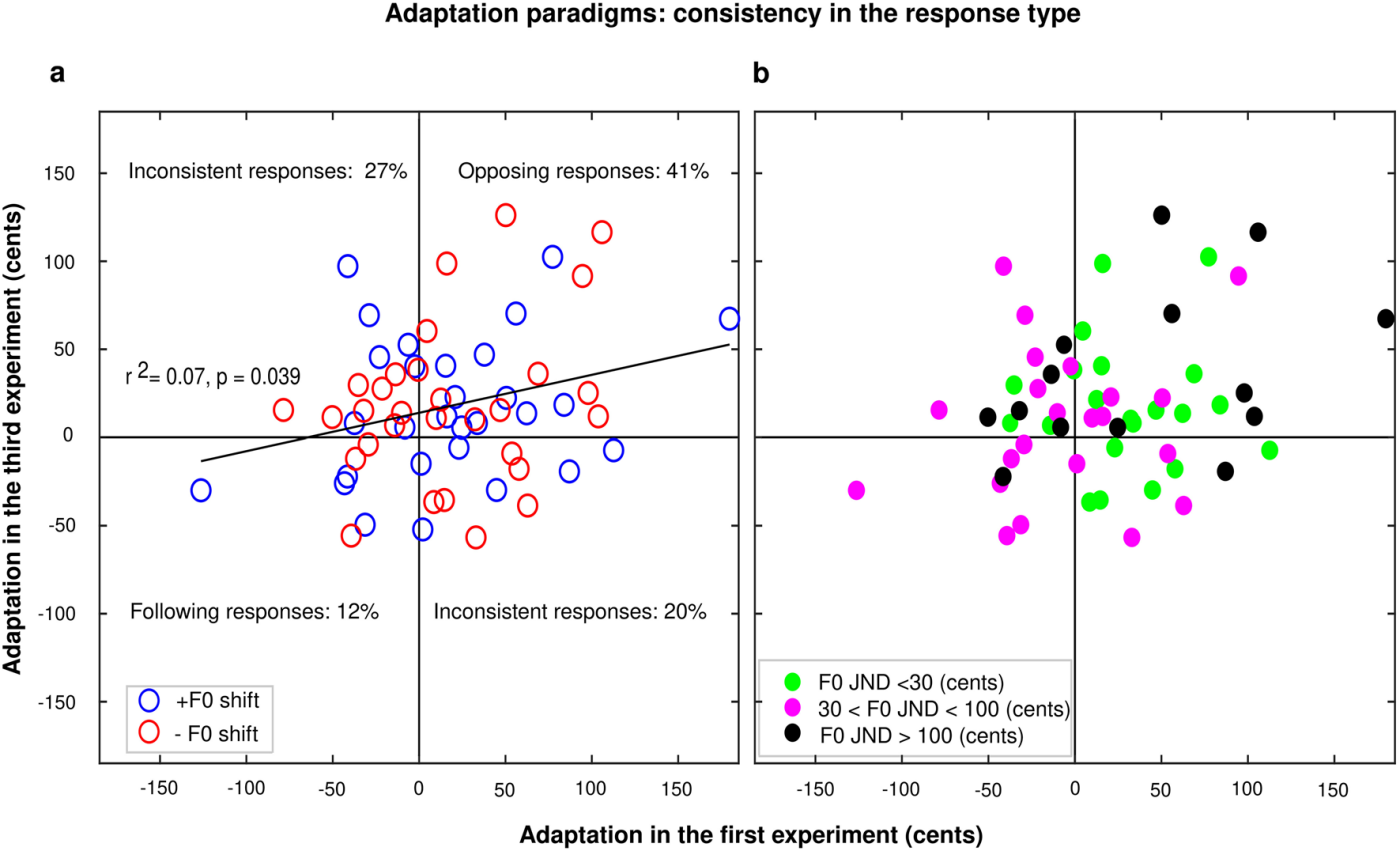
(**a**) Four-quadrant graph illustrates different types of consistent and inconsistent vocal behavior of participants, within the first 100 ms of productions, (**b**) The same data, with individual participants colored by their sense of pitch.

To have a deeper insight into the variability of the outcomes in each experiment, we examined the standard deviation of the adaptation magnitude (measured from the early-F0 metric) across participants in the first and third experiments: it was 53.6 cents in the first experiment and 43.0 cents in the third experiment. Thus, even though our personalized paradigm presented errors that were more likely to be perceived as self-generated, we failed to (sufficiently) homogenize the outcomes across participants, and must therefore reject the hypothesis that the personalized paradigm would “incite” participants to behave in a more consistent manner, typically opposing. Interestingly, 36 out of the 59 participants exhibited an opposing response in the first experiment and 40 in the third one. Considering the type of responses as a test/retest reliability exercise, we found a significant correlation between the two experiments (r2 = 0.07, *p* = 0.039) (see left panel of Fig. 4). This is reassuring to some degree, given that over half of the participants were consistent in their responses between the two experiments: 41% exhibited consistently opposing behavior, 12% showed consistently following behavior; in contrast, 20% showed opposing responses in the first experiment and following responses in the third one, and 27% showed following in the first and opposing responses in the third experiment (see discussion).

### Is adaptation dependent on F0 JND?

**Figure 5:**
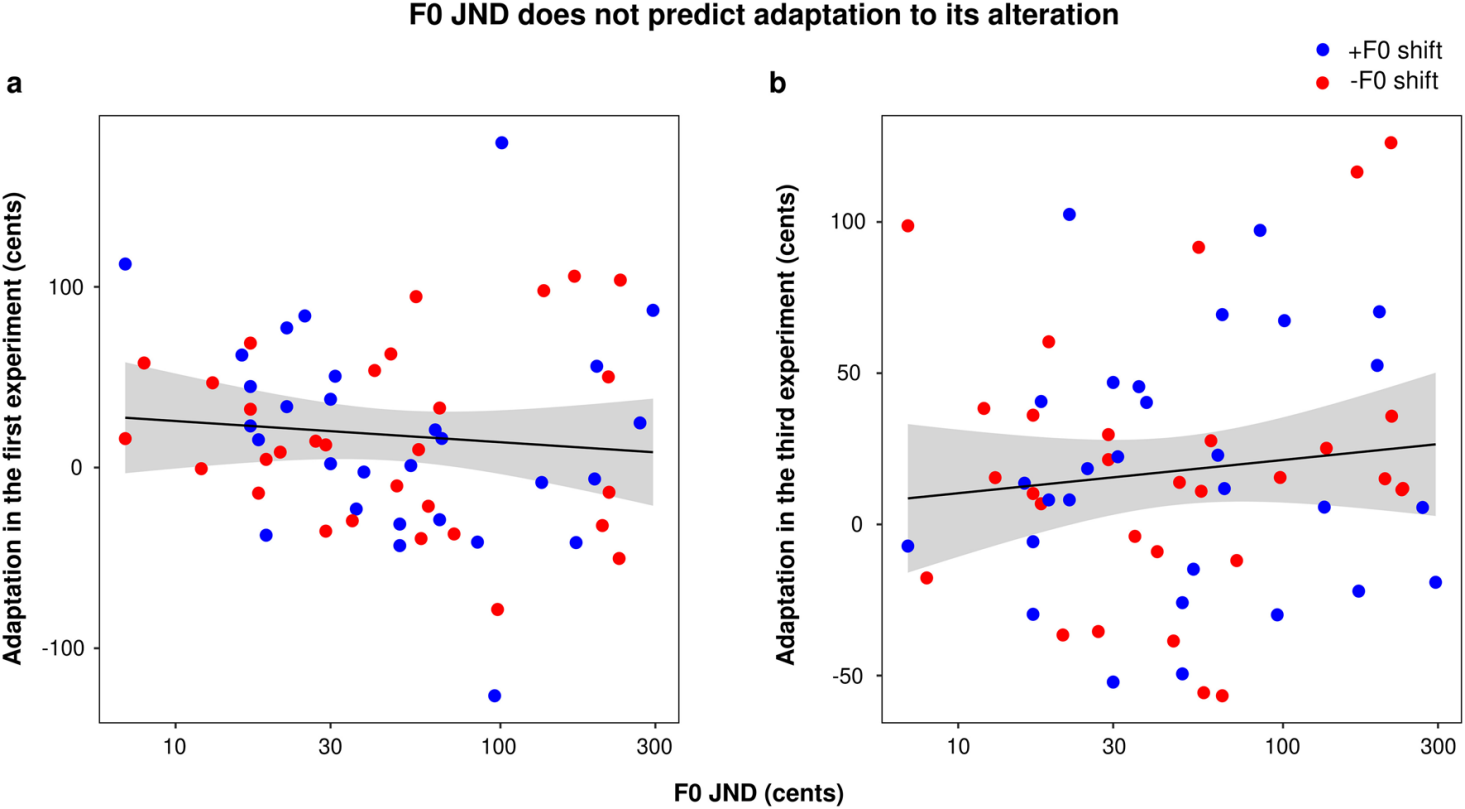
Lack of relationship between F0 JND and the magnitude of adaptation in the first experiment (**a**) or the third experiment (**b**).

One important question at the heart of this study was whether the amount of adaptation in response to an F0 alteration depended on the participants’ F0 JNDs. In the first adaptation paradigm, there was no such relationship at all (r2 = 0.01, *p* = 0.478, again expressing JNDs on a log scale), not even when considering vowel-specific JNDs and vowel-specific adaptation (vowel/a/: r2 = 0.03, *p* = 0.181; vowel /e/: r2 = 0.01, *p* = 0.489; vowel /o/: r2 < 0.01, *p* = 0.902). Likewise, in the personalized adaptation paradigm, we found no significant correlation between F0 JNDs and the adaptation magnitudes (r2 = 0.01, *p* = 0.406, again expressing JNDs on a log scale), nor between the vowel-specific JNDs and vowel-specific adaptation (vowel/a/: r2 < 0.01, *p* = 0.813; vowel /e/: r2 = 0.02, *p* = 0.308; vowel /o/: r2 < 0.01, *p* = 0.728) (see Fig. 5). In other words, the present results rejected the idea that adaptation magnitude depends on F0 sensitivity. Note that we also examined the significance of the null hypothesis^44^ (see Supplementary Information 1), and the present data allowed us to draw sufficiently convincing evidence for the *absence of this relationship*.

A related, but tangential, concern was whether the consistency in the type of response (e.g., consistently opposing, or consistently following) could depend on F0 sensitivity, even if the magnitude of the effect was unrelated to it. To address this, we divided participants into three groups, based on their F0 JNDs. Twenty-one participants had JNDs below 30 cents, 24 participants had JNDs between 30 to 100 cents, and finally, 14 participants had JNDs above 100 cents. The right panel of Fig. 4 illustrates the same data as in its left panel (discussed above) but with individual participants colored by their sense of pitch. We performed a mixed ANOVA with one between-subject factor (group, based this time on F0 JND) and one within-subject factor (experiment) to examine whether belonging to one group or another favored consistency in the type of response, i.e., being located within one quadrant of this correlation space between the first and the third experiments. There was a main effect of group [F(2,56) = 5.1, *p* = 0.010], no effect of experiment [F(1,56) < 0.1, *p* = 0.893], and no interaction between them [F(2,56) = 1.0, *p* = 0.379]. The effect of a group arose because participants with intermediate JNDs (30 < JND < 100 cents, pink symbols) behaved in a more erratic manner than the two other groups: (*p* = 0.011) compared to the participants with poor pitch sensitivity, and (*p* = 0.111) compared to the participants with fine pitch sensitivity, while the two other groups did not differ (*p* = 0.821). In conclusion, there was no straightforward relationship between F0 JNDs and the consistency in the responses (or at least, its interpretation is not trivial).

## Discussion

The purpose of monitoring voice pitch is to gain better control of phonation and intonation within a sentence to transmit linguistic and affective cues, with the least ambiguity, to the receiver. It is thus a critical tool in establishing effective communication, with apparent consequences at different developmental stages. Auditory feedback is believed to be one of the primary sources required for this monitoring. In the present study, we performed two separate adaptation experiments to explore whether a personalized size of the F0 shift would have led to more consistent or more stable opposing responses and fewer following responses than a fixed non-specific size such as one semitone, thereby accounting for some of the inter-subject variability notorious of such paradigms (from past studies). Results confirmed that repetitive presentation of F0-shifted vocal stimuli in a constant and predictable manner led to sensorimotor adaptation in both experiments, regardless of the direction of the F0 shift, but overall it was very short-lived once the F0 alteration stopped (and in fact completely absent in the first experiment). These results are partly in line with previous reports^4,5,17,22^. However, we found no link between the magnitude of this adaptation phenomenon and the sensitivity to the error (F0 JND), despite our best effort to optimize the efficiency of the paradigm and inciting participants to believe that the error was self-generated (since it was, precisely, just-noticeable for everyone).

### Adaptation or compensatory response?

When a manipulation, either in F0 or in formants, is applied on auditory feedback, the immediate vocal responses, within the first 100 ms time window, are believed to reflect the stored motor plan^45^. However, the vocalizations after this time window represent a mixture of the reflexive vocal responses to the perturbations (i.e., compensatory responses), and adaptive changes. The reflexive responses are believed to be driven by a neural monitoring system with the aim of stabilizing the vocal F0^31^. On the contrary, the adaptive changes arise following the error detection process, where the speech monitoring system detects some conflicts and errors by comparing the predicted feedforward plan with information coming from auditory/somatosensory feedback controllers. Indeed, in any functional system, error arises when performance and its outcome go in different directions, instead of overlapping. Therefore, the control system attempts to solve the conflict^46^ by refining the feedforward plan through learning from sensory feedback information, a process known as sensorimotor adaptation^47^. Herein, the somatosensory system is faster, sending information to the speech monitoring system with a 20–75 ms delay^48^, whereas the auditory system takes 80–120 ms^6,49^ to process the vocal output and inform the control system. Therefore, because feedback-based corrections take more than 100 ms to begin, the first 100 ms gives a cleaner measure of feedforward control and, thus, adaptation.

**Figure 6:**
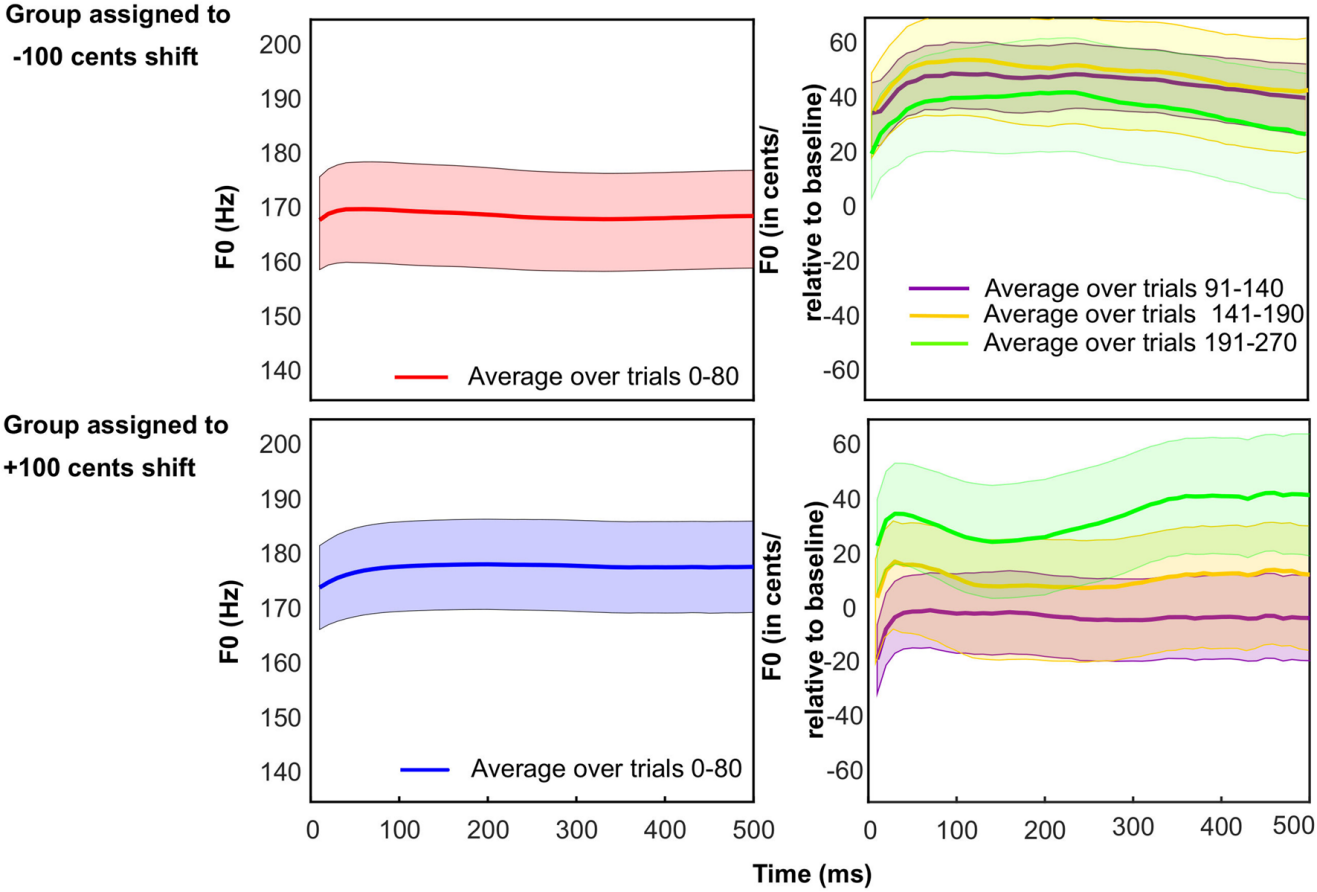
Mean F0 trace for baseline trials (intact feedback) averaged across participants in each group (left panels). Mean F0 trace for hold-phase trials (feedback shifted by ± 1 semitone) or for washout trials (feedback intact once again) across participants in each group (right panels).

To examine these processing steps thoroughly, we looked at the first 500 ms of vocal responses, recorded during the first experiment of the current study. We first averaged F0 traces across the 80 baseline trials, to provide a single mean trace per participant. As can be seen in the left panels of Fig. 6, there were slight curvatures at the beginning of the production, but the rest was very stable for these baseline trials. To examine the strategy of the speech motor system in updating its motor plans under constant F0 shifts, we reiterated the above-mentioned process for the first and second halves of the hold phase and the washout trials but expressed each mean trace in cents relative to the averaged baseline trace of each participant (i.e. more similar to the traditional method for expressing F0 data relative to baseline). The right panels in Fig. 6 show that there were again slight curvatures for the first 20–30 ms. Past 30 ms, the group assigned to downward F0 shifts (top-right) exhibited a fairly stable production up to about 250 ms, followed by a slight trend downwards. The group assigned to upward F0 shifts (bottom-right) exhibited an opposite curvature, although this did not occur in the first half of the hold phase and was more pronounced for the washout trials. These slight curvatures might represent possible compensatory changes, but they are certainly minor. In other words, there was overall little evidence for compensatory influences. This conclusion is corroborated by the fact that results aforementioned were very similar whether they were based on the first or middle 100-ms segments (see Figs. 1 and 3). Finally, note that the patterns shown in Fig. 6 illustrate the limitations of expressing F0 data relative to baseline trials: due to slight upwards trends in mean F0 across the entire experiment, the amount of adaptation would be largely overestimated in the group assigned to a downward shift, and underestimated in the group assigned to an upward shift, hence the need for a better normalization method that takes into account some of the latest washout trials.

On the other hand, an important expectation from an adaptation paradigm is the after effect, which generally supports the idea that learning of a new motor plan (or remapping) has occurred. We did not find any evidence for after-effect, except for a very short-lived effect in the very first block of the washout phase and only in the personalized paradigm. One potential cause of the lack of any after-effect here was that speech motor plan for controlling F0 updated very rapidly. The maximum adaptation in the first block of the hold phase (in fact occurring over the ramp) supports this claim and demonstrates that feedback-based corrections happened as soon as the error perceived. This may explain why evidence for this learning lasted only for a short period of time, i.e., during the first 8 trials the washout phase, but was lost once averaging over the first 30 trials of the washout phase. Furthermore, Bayesian statistics provided moderate evidence that there was genuinely no after-effect (within these 30 trials) in this study (see Supplementary Information 1). This null hypothesis is rather consistent with the progressive reduction in the adaptation effect throughout the hold phase. To our knowledge, however, this pattern has not been reported so far, and so, it calls the nature of the response (adaptive or compensatory) into question.

### Adaptation stability over hold phase trials

We found that opposing responses to a fixed size of F0 shift, i.e., ± 100 cents, were more robust in the first half of the hold phase. By examining the personalized paradigm, we found that this attenuation pattern was more prominent for the group assigned to upward F0 shifts. Attenuation in the magnitude of adaptation over the trials of the hold phase was in contradiction with the study by Behroozmand and Sangtian^29^, Abur et al.^42^, and Murray and Stepp^22^.

To account for these apparent discrepancies, we propose three interpretations. First, given the large inter-subject variability reported in adaptation paradigms, the aforementioned studies could have happened to recruit well-behaved participants, facilitated by their smaller sample size. The number of our participants was more than twice the sample size in the study by Abur et al.^42^, three times that of Murray and Stepp^22^, and five times that of Behroozmand and Sangtian^29^. Besides, we made no attempt to eliminate potential outlier participants based on their outcomes in the paradigms.

Second, the type of vowels could have produced different effect sizes across these studies. This interpretation is largely supported by the finding that, in the first experiment, the adaptation magnitude was larger with vowel /a/ than vowels /e/ and /o/ (when looking at the middle F0 segments). More importantly, the choice of a single vowel type might perhaps facilitate motor recalibration and the observation of larger adaptation magnitudes. In our study, it is possible that switching vowels at random from trial to trial led to increased demands (potentially attentional effects) into which motor command needed to be readjusted, eventually leading to reduced adaptation magnitudes (at least for vowels /e/ and /o/).

Since we clarified that F0 JNDs were similar across groups and vowels, we speculated that there might be some articulatory explanations to the “vowel /a/ effect” in the first experiment. For example, singers often fall back on a large mouth opening (like /a/) when training to control their vocal cords^50^. We measured the within-trial standard deviations of F0 of baseline trials in vowel /a/, compared to /e/ and /o/ and found slightly lower values for vowel /a/ than two other vowels (first experiment: vowel /a/ = 37.9 cents; vowel /e/ = 38.6 cents; vowel /o/ = 39.7 cents, and third experiment: vowel /a/ = 40.0 cents vowel /e/ = 41.5 cents; vowel /o/ = 41.3 cents). A mixed ANOVA with two within-subject factors (experiments by vowels) revealed no main effect of vowel [F(1.8,102.1) = 1.9, *p* = 0.160], and no interaction between vowels and experiments [F(1.7,101.0) = 0.9, *p* = 0.394]. So, vowel /a/, /e/ or /o/ had similar within-trial variability in F0. Alternatively, we speculated that these differences might have something to do with the intensity at which vowels were produced. To address it, we extracted the intensity of trials of the baseline and hold phase of both experiments. Results (see Supplementary Information 2) showed that vowel /o/ was the loudest, followed by /a/, and /e/ was the weakest. Given that adaptation magnitude was similar for /o/ and /e/, we conclude that direct comparisons between vowels are not meaningful with respect to adaptation magnitude. However, there was an intriguing interaction between vowel and phase (see Supplementary Information Fig. S1), reflecting that vowel /a/ was the only vowel that significantly increased in intensity (but arguably a weak effect size, only 0.7 dB between the baseline and the hold phases), similarly across the two experiments. Thus, relative changes in intensity within a given vowel might be something to explore further in the future. This being said, in this intensity analysis, there was no main effect of group, nor any interaction with group and thus, the different behavior of /a/ could not be explained by between-vowel differences in intensity. Note that, given these findings, intensity could not explain either why adaption was weak in the second half of the hold phase of the group assigned to the upward F0 shift.

Finally, a third interpretation—as to why our findings were distinct from past studies (particularly in how adaptation faded away throughout the hold phase)—is that during the second half of the hold phase, the speech monitoring system could have continuously optimized the stored motor plan with a series of successive refinements. We call them “optimized” plans because the progressive loss of adaptation combined with the absence of after-effect suggested that it is neither the first stored motor plan nor its updated versions that directed the fast adaptation within the first trials of the hold phase. The observed decrement in the magnitude of adaptation responses could be due to a repeated exposure to the pitch-shift stimuli. This habituation to auditory stimuli, which is not a new idea^51^, has been reported in numerous studies and is considered as an expected behavior from speakers while facing predictable pitch shifts^33,52^.

However, we believe that continuous attenuation in the magnitude of adaptation was not a function of habituation effect per se. Indeed, the long-term recalibration of motor plans might be driven by factors other than sensory-error-based adaptation. Most relevant to our study, we believe that the type of the vowels that we used possibly intervened in the optimization process and imposed some degree of adjustments to this series of continuous motor command tunings. This means that switching fast between three vowels instead of keeping one articulatory gesture^53^ could interfere with the consolidation of an updated motor plan. This interference might be the reason behind the interactions of vowel by group in the washout phase of our results. These new articulatory gestures, not in line with the habitual positional status of muscles, might impose fatigue on the laryngeal muscle spindles. An alarming signal might then inform the system of an excess of energy consumption, in contradiction to the gold standard of the system (maximization of function while minimizing energy consumption^54^). This might force the entire system to seek for the highest level of stability while keeping its best function. Meanwhile, the auditory system continues to send auditory information relevant to its current status to the monitoring system, but this information would become less important and deceptive for the speech motor control system^14^. Thus, the system might apply more weight to a reliable information source like somatosensory feedback system^54^, especially since this feedback conveys detailed information of vowel types and their categories^55^.

We suggest that this series of continuous transitions from the stored motor plan to successive recalibrated versions (e.g., the first F0 jump in the opposing direction) and finally to the most optimized versions (e.g., the attenuation of the adaptation magnitude) is what Figs. 1 and 3 reflect. To summarize, our results suggest that the speech monitoring system could continuously refine itself with respect to energy consumption and fatigue, attempting a number of motor plans to reach a maximum F0 stability at the lowest cost. This being said, switching between vowels may not be only detrimental to the quality of this paradigm. At least from a speaker/participant’s perspective, having to produce different vowels and maintain attention on the monitor to know which vowel to produce next seemed to have made for a more interesting task.

### Inter-subject variability in vowel production led to a wide range of F0 JNDs

Following on previous findings^22,30,39^, we had hypothesized that the inter-speakers’ variability in response to F0 perturbation would be due to the broad range of F0 JNDs across different speakers, but we failed to find direct evidence for this hypothesis. Similar investigations have been conducted to directly highlight the mechanistic link between the ability to detect F0 changes and compensate for them. For example, Scheerer and Jones^56^ developed a smart design of a compensation paradigm, by asking participants to vocalize vowel /a/ for 130 times while experiencing unaltered, and F0 manipulated feedback with six occurrences of F0 changes per vocalization (necessarily requiring a brief 200-ms duration for each perturbation). The authors down-shifted the F0 with different sizes of either 5, 10, 15, 20, 25, 30, or 40 cents (but the same size on a given trial). After each trial (7-s long), participants had another 7 s to decide whether they detected a manipulation in their voice pitch. After performing this “active paradigm”, the authors asked their participants to passively listen to their vocalizations and judge whether they detected pitch manipulations. The smallest F0 shift that elicited compensatory responses during vocalization was 10 cents, with a linear increase in the magnitude of responses from 5 to 40 cents of the F0 shift. However, the sensitivity threshold of participants toward F0 changes in the passive listening task was *poorer* than during the compensation task (active vocalization), with the smallest threshold equal to 15 cents. Scheerer and Jones^56^ argued that the 5 cents difference between the active compensation and passive detection might be due to the involvement of the somatosensory feedback in the active condition and pointed out that inherent variability in the recordings (like in our study) made the perceptual task more difficult when no complementary cues were provided from other modality. Our findings are exactly in line with their conclusion (bottom panels of Fig. 2).

In a similar concept, but a different design, we first measured the F0 JNDs of our participants toward their own production, and then used these values as the size of F0 alterations in a new round of adaptation paradigm. Having 210 trials of a 3I-2AFC task, we found an average JND of 47.7 cents, with a broad range from 7 to 295 cents. This sense of pitch might be surprisingly large compared to the fine sensitivity known for computer-generated harmonic complexes being often less than 1% (or 17 cents, e.g. ^57–60^.), or compared to F0 JND of 28 cents that Murray and Stepp^22^ reported for a 500-ms sustained vowel /ɑ/ production recorded from a child. Yet, this apparently rougher sensitivity also has merits compared to any of the aforementioned studies because it encompassed the sort of spectro-temporal variations that occur during human vocalization, even within 300 ms, and included different forms of roving (mean F0, level, spectral envelope) that are specific to each participant’s vocal apparatus. Indeed, there is considerable variability between individuals in laryngeal structure and their effect on the speaker’s voice characteristics. We suspected that variability inherent to the recordings of the first experiment was the primary source of this poorer range of F0 JNDs. If the vocal cords of some participants were unstable, it seems only fair that these participants would have had difficulty recognizing changes to their own voice pitch. This is not a particularly novel finding: differences between intervals of the forced-choice task in terms of loudness or timbre percepts are known to hinder pitch sensitivity^61,62^.

By carefully examining the participants’ productions of the baseline trials (used exclusively for the F0 discrimination task), we found support for this interpretation: F0-variability inherent to the recordings generated some distraction that elevated JNDs in some participants. In other words, participants who had less control over their vocal cords were less sensitive to F0 differences in the 3I-2AFC task. This being said, the positive correlations between the standard deviations of F0s across and within trials and the F0 JNDs should be taken with caution. There are different sources of F0 variability at multiple time scales (e.g., pitch drift, vocal tremor, vibrato), and they would not affect the 3I-2AFC task equally. Indeed, the Praat software monotonized the F0 contours. This ought to nullify the impact of these sources, unless they had some collateral effect on other aspects of production, as in less stationary temporal envelopes and more fluctuating timbres. Finally, note that this procedure measured F0 sensitivity in conditions where participants listen passively, whereas ideally, one would wish to measure this sensitivity in the same conditions as they occur during the adaptation paradigm, i.e., participants actively speaking and listening to their slightly delayed feedback. But doing so would impose some constraints into the amount of roving (level, mean F0) one can inject in the task for the JNDs to accurately represent F0 coding^63^.

### Auditory sensitivity is an important factor for error detection but is not the sole player for error correction

In the last experiment, we expected to observe a more homogeneous adaptation magnitude across participants because this paradigm respected the concept of familiarity/self-identity, i.e., it optimized participants’ belief that they heard their own voice and not an alien one. Our results partially confirmed this expectation by showing that at least a small but measurable after-effect could be found in the first few trials of the washout phase, and the adaptation magnitude over the hold phase was as great as in the first experiment, but the results failed to support more homogenized responses. Moreover, adaptive responses were elicited by alterations as small as 7 cents (slightly less than those reported in Scheerer and Jones^56^) and by alterations as large as 295 cents, with no striking loss of adaptation in the large shifts.

Furthermore, through the test/retest exercise aforementioned (see Fig. 4), we found that 41% of participants exhibited consistently opposing behavior, regardless of the size of the shift; while 12% of participants showed consistently following behavior, and all except one had F0 JNDs smaller than 100 cents. This result somehow rejects the idea proposed by Korzyukov et al.^36^ as clearly, people can exhibit following responses even when making sure that they believe it is their own voice. For instance, 20% of our participants showed opposing responses in the first experiment, and following ones in the third experiment, and yet, nearly all (except one) had JNDs less than 65 cents.

Adaptation magnitude did not relate to F0 JNDs in either experiment, and the consistency in the response did not relate simply to F0 JND either. The participants with the poorest sense of pitch presumably did not realize that their voice pitch was being manipulated, i.e., they should have believed it was their own voice, and many exhibited opposing responses. However, this was also the case of participants with the finest sense of pitch, for whom a shift of 100 cents was large enough for their brain to realize it was not self-generated. Perhaps, an individual case would illustrate this point further. A few participants informally reported that their voice was somehow manipulated (without being able to tell it was their pitch); this was the case of someone with an F0 JND of 7 cents, who exhibited a great opposing response to the 100-cents shift and nonetheless showed a following response in the third experiment. Experiencing a shift that is perceived as "self-generated" is not enough to induce an opposing behavior.

In both experiments, age had no effect as the correlations between the magnitude of adaptation and chronological age in the first, or the third experiment failed to reach significance. These results, which are in line with Liu et al.^64^, are perhaps another indication that F0 sensitivity and adaptation are independent**.**

If this is so, a reasonable conclusion could be reached by considering the error detection and error correction as two separate, yet interconnected, processes. Indeed, the two systems should have reciprocal connections to perform a well-organized process of error detection and correction successfully. A very recent study^43^ reached the exact same conclusion. The speech error detection system has a complex nature with many subcomponents involved, and likely under the supervision of shared sensorimotor brain areas responsible for the functional coupling between speech perception and production systems^65^. Nozari et al.^46^ believed that a generic monitoring system detects all the conflicts in the speech output based on the comparisons that this system makes between the actual movement and a possible reference, like a feedforward plan of the same token (i.e., predictions of the system). Taken together, it seems that the auditory system helps with error detection but does not play a primary role in this process^47^. Therefore, the sensitivity to one’s own voice pitch is only one component in the equation that works as a subcomponent for error detection.

## Future directions

One reason for designing this study was our clinical interest in examining the vocal behavior of cochlear implant (CI) users in response to F0 manipulations, in the future. CIs provide a poor spectro-temporal resolution, and complex pitch coding is severely degraded^66,67^. As a consequence, their users have poor F0 JNDs, on the order of 2–3 semitones even for users implanted early in life^68,69^. It is not clear whether CI users can incorporate auditory feedback with feedforward plans to monitor their speech output. A recent study^70^ showed that CIs might help their user to refine their internal motor commands, but voice pitch monitoring might not happen regularly. The main message of the current study is that a high auditory sensitivity does not guarantee a successful error correction in an ongoing vocalization. In other words, the goal of CI manufacturers and clinicians should not be uniquely focussing on pitch perception enhancement. A third party, like a speech therapist, audiologist, or primary caregiver in the case of CI children, could be incredibly powerful in relaying the error information to CI users (or more generally voice intonation in diverse contexts), information that their device is not sufficiently good at delivering. This might, over time, help CI users to rely less on somatosensory feedback.

## Conclusion

The present study hypothesized that variability in the sensorimotor adaptation might be due to different sensitivity toward detecting the errors. However, our results did not support this hypothesis as there was no relationship between F0 JNDs and adaptation magnitudes in either experiment (fixed F0-shift or personalized F0-shift). Furthermore, the personalized paradigm did not encourage more people to show opposing responses, even though it was effective (since the effect size was similar to that of the traditional paradigm). This efficiency was, however, likely the result of the smaller shift experienced in the personalized paradigm.

Taken together, the adaptation phenomenon observed in this study is evidence for continuous control of speech, which happens whether the size of the F0 shift is subtle or very salient^30,71,72^. However, it is critical to consider the responses of this paradigm as a combination of adaptive processes, motor plan refinement, and most importantly, systemic optimization imposed on the entire system^73^.

Our results nourish a previous assumption that multiple internal sources are recruited within the perception-production loop, and assessing the involvement of each effector system is not a trivial enterprise. The apparent gap between theoretical perspectives (i.e., proposed models on speech motor control) and clinical demands, especially for speakers with hearing problems who might have an unbalanced feedback-feedforward loop, highlight a need for more studies to translate an intricate picture of the subsystems involved in speech monitoring to the daily life. Filling this gap helps not only the hearing aid/CI device developers but also clinicians who work in speech rehabilitation to enhance clinical interventions and optimize the desired outcomes. There are to date still important debates about methodological issues on well-known laboratory paradigms such as adaptation to altered feedback and our innovative approaches may help solve some of them while maintaining a strong ecological relevance to speech perception and production.

## Methods

### Participants

Sixty adults from ages 19–72 (mean age: 32.9 ± 10.3 years, 32 female) participated in this study. None had neurological and hearing problems. As an inclusion criterion, all participants had normal hearing as determined by pure-tune audiometry at octave frequencies from 125 to 8000 Hz^74^, with air-conduction thresholds < 20 dB hearing level (HL). Two participants had formal singing training, but the rest were non-musicians. One participant was excluded from the study because of high level of jitter in his voice while producing vowels. This study was approved by the Institutional Review Board of the Faculty of Medicine at McGill University under the principles expressed in the Declaration of Helsinki. Informed written consent was obtained from participants before their involvement in the project.

### Experimental design

All experimental procedures were conducted in a sound-attenuated booth. The study consisted in three experiments. In the first experiment, we asked participants to perform an altered auditory feedback paradigm whereby an error in F0 was induced in real-time while the participants were vocalizing. In the second experiment, we measured the F0 JNDs of participants to their own voice pitch using a three-interval two-alternative forced-choice (3I-2AFC) paradigm. And finally, in the third experiment, we replicated the first task, but personalized the size of the F0 shift by adjusting it based on the F0 JNDs that we measured in the second experiment. All experiments were done consecutively in this order, without breaks, within about an hour. The paradigms are further detailed below.

### Adaptation paradigm

**Figure 7:**
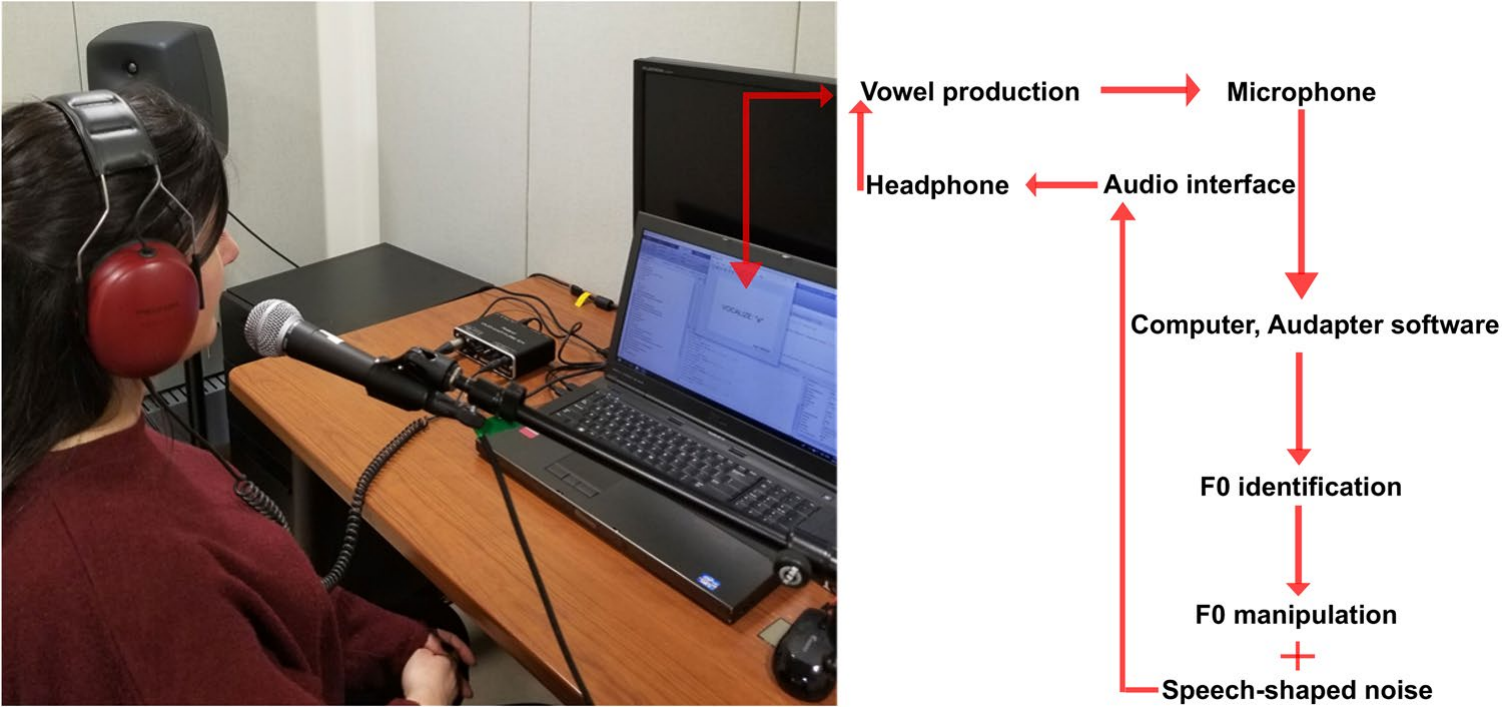
Experimental setup: vocalization was captured by a microphone and fed to the computer through an audio interface. Each vocalization was analyzed and re-synthesized in pseudo-real-time using the Audapter software. The manipulated signal was mixed with speech-shaped noise and then fed to the individual via over-the-ear headphones.

Participants produced 270 vocalizations of three different vowels /a/, /e/, /o/ while hearing their own voice through headphones. These vowels were chosen to remain within the native phonetic space of the participants^75^ and included examples of close or large openings (/o/ or /a/, respectively), factors that might influence phonation and the ease with which vocal cords are controlled. Participants were instructed to vocalize these vowels, which were randomly presented on a screen in front of them, immediately after seeing the relevant cue (setup depicted in Fig. 7). Each visual cue containing the name of the vowel remained on the screen (Dell Precision laptop with a 17″ screen) for 1.5 s, followed by half a second pause in between.

The vocalizations were captured using a Shure SM58-CN microphone (5–7 cm away from mouth), fed to the computer through an audio interface (DUO-CAPTURE EX Roland Corporation, Hamamatsu Kita-Ku, Shizuoka, Japan), and recorded with a sampling frequency of 48 kHz (later down-sampled to 11.2 kHz). Each vocalization was analyzed and re-synthesized in pseudo-real-time using the Audapter software^76^. The F0 of the vocalization was estimated and subsequently shifted up/downward or left intact (but still reprocessed based on the same routine). The manipulated signal was then mixed with speech-shaped noise to reduce the amount of bone-conducted acoustic feedback (with an intensity continuously updated at 3 dB less than the participant’s voice, until voicing was no longer detected). The feedback signal was fed to the individual via over-the-ear headphones (3.5 mm stereo, Peltor workstyle, Type HT7A-30GP) with less than 25 ms of delay, similar to our previous study^77^. This delay represents the time needed for three processing levels including the signal input to the audio interface, processing of the audio signal, and finally audio output from the interface to the headphone^78^.

**Figure 8:**
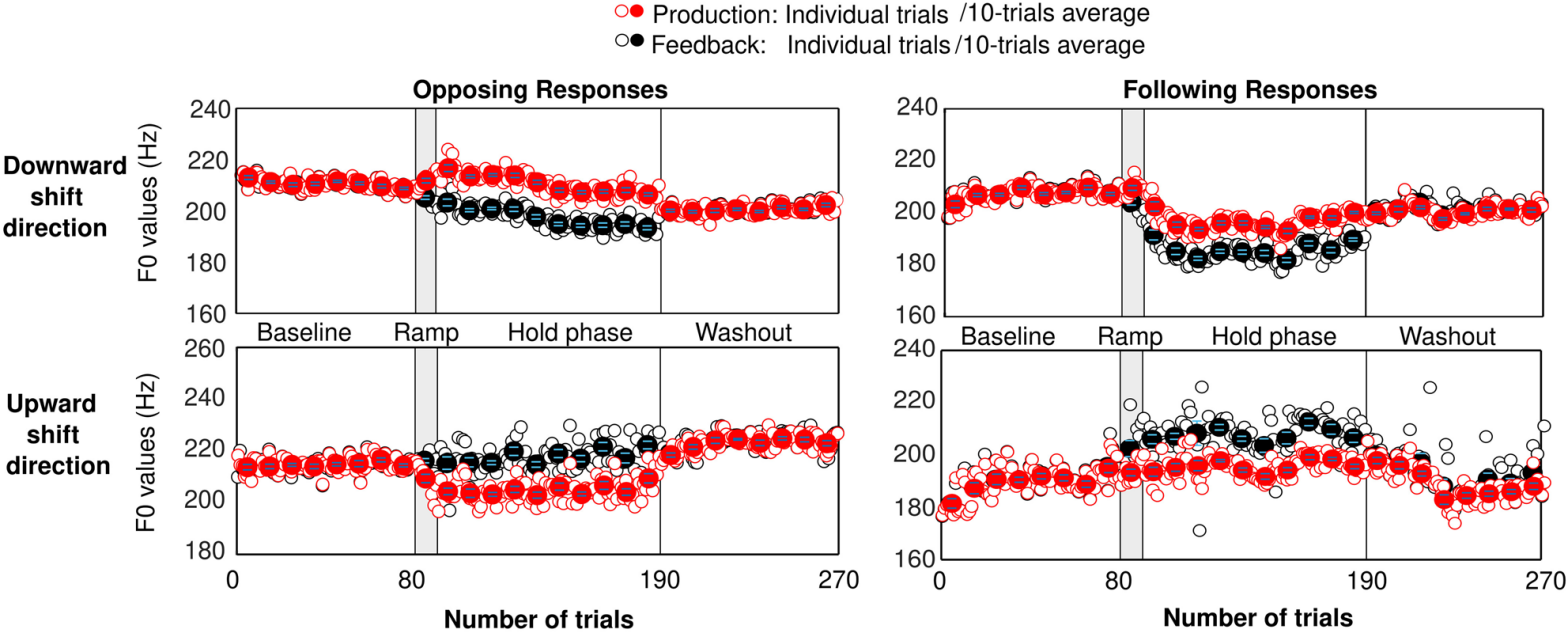
Samples of vocal behavior taken from four speakers in response to the auditory feedback manipulation. Speakers experienced downward (top panels) or upward (bottom panels) F0 shifts in their auditory feedback. During the baseline condition, the auditory feedback was intact. By the beginning of the ramp phase, the pitch of feedback channel was gradually shifted until it reached a value of 100 cents and then was held constant in the hold phase. The auditory feedback was intact during the last 80 trials of the experiment (washout).

In line with the typical adaptation paradigms^73^, F0 manipulation was carried out over four distinct phases. In the first phase called the Baseline, participants received unaltered auditory feedback of their vocalizations for the first 80 trials. This condition was essential to obtain a reliable estimate of the mean F0 of each participant under normal speaking/listening condition. In the second phase called the Ramp, the voice’s F0 of the participants was gradually shifted during the next 10 consecutive trials up to 100 cents for the first experiment and up to F0 JND for the third experiment. The gradual change was implemented as an attempt to make the F0 shift unnoticeable by participants. The F0 shift was applied at vocalization onset and kept constant as long as the participants vocalized. Participants were assigned randomly to two groups experiencing either upward or downward F0 shift, but consistently across trials and across first/third experiments. In the third phase called the Hold phase, covering the next 100 consecutive trials, the F0 shift was maintained constant at the same value (100 cents or JND) in the same direction (sign). In the fourth phase called Washout phase, F0 was returned to its unaltered value for the final 80 trials. This condition is traditionally used to assess the extent to which changes in F0 production persist over time. In our case, however, we used it also to better define the reference F0s (see data analysis). As illustrations, four examples of production data along with their respective feedback channel are depicted in Fig. 8. The top and bottom panels illustrate the vocal behavior of speakers who experienced downward or upward pitch shifts in their auditory feedback. Notice two different behaviours in the graphs, as the left panels delineate the (more typical) opposing responses, while the right panels show the (more occasional) following responses. Note that the auditory feedback contained speech-shaped noise mixed with the manipulated production, so F0 estimates were less accurate than for the production channel (hence the slight differences between red and black symbols even in the absence of F0 alteration).

Before the experiment, we explained to participants that they might hear some sort of echo in their auditory feedback and asked them to keep their voice at a comfortable loudness during their production. They were neither told to match their voice pitch with the heard vowel nor to ignore the auditory feedback entirely. Participants were instructed to vocalize each of the vowels requested from the monitor prompt, but it happened that participants missed a trial, for example by coughing during a vocalization or mistiming their production. All trials were thus screened through visually by the first author to ensure they contain continuous vocalization and rejected otherwise, thereby excluding them from any further analysis. Two rounds of the data analyses were performed for each dataset. After finishing the first round, the first author visually inspected all the individual trials of the participants and then removed bad trials from the dataset, including the trials with zero production, trials with apparent effect of coughing on them, and trials with less than 400 ms duration of the stable portion of the vocalizations. At the end of the second round, on average, 0.7% of trials in the first experiment and 0.5% of trials in the third experiment, mostly from the baseline trials, were removed from the data, and the results of this study are reported based on the clean data.

### F0 JND measurement

The perceptual task was concerned with the participants’ ability to detect changes in their own voice pitch, not just how sensitive they were to the pitch of complex harmonic tones. For this reason, we wished to use recordings of their own voice. The previous experiment had recorded simple productions of vowels that were intentionally sustained over time and were consequently deemed adequate to serve as experimental stimuli in this task. Only the 80 baseline recordings were selected to avoid any unexpected production behavior that could have resulted from the adaptation phenomenon. In each trial, a recording was picked at random among the 80 choices, i.e., it could be any of the three vowels. The Hilbert envelope was extracted from the 1.5-s long signal and cut at 10 dB below the peak amplitude on either side in order to remove any portion of the recording that had little voicing. Within this voiced window, a random onset was selected, and the standard deviation of the envelope level was calculated over the next 300 ms. If this standard deviation exceeded a fixed criterion of 3 dB, the stimulus was deemed insufficiently stationary, and another random onset was searched within the voiced window. This procedure continued until a 300-ms long portion satisfied the criterion of pseudo-stationarity (level fluctuating by less than 3 dB), for a maximum of 10 iterations. In cases where the search failed after 10 iterations, a new recording was chosen among the 80 possible choices, and the same procedure was reiterated. This is important because performance in a pitch discrimination task can easily deteriorate as intervals differ in loudness or timbre percepts ^61,62^. Thus, while we wanted to use ecological stimuli, we also wished to limit as much as possible uncontrolled acoustic variability within the stimuli.

The 300-ms signal was then gated with 30-ms onset/offset cosine ramps and sent to Praat^79^ for flattening the F0 pattern throughout, at a given F0 value. Two intervals had the same F0, and one (the target) had a slightly higher or lower F0. The silence duration between intervals was set to 300 ms as well. Finally, the three stimuli were recalibrated at 65 dB SPL and presented in silence with a ± 3 dB level roving. It may seem odd to have gone through the trouble of choosing a pseudo-stationary signal since stimuli were eventually roved by ± 3 dB. This was done in an effort to control the amount of variability injected in the task in order to make unwanted cues—here, subtle changes in loudness induced by F0 differences—unreliable^80^. In the same logic, the reference F0 was roved across trials between 150 and 200 Hz to discourage participants from relying on particular values of F0 that could have resonated more or less with the participant’s vocal tract. Note that this range was identical for male and female participants. Stimuli were sampled at 48 kHz with a resolution of 16 bits and were presented via headphones to participants.

The test followed a Bayesian adaptive method^81^. Rather than a standard staircase, where each trial is determined by success or failure on the previous couple of trials with predetermined step sizes as a function of reversals^82^, the Bayesian approach aims to maximize the gain of information by constructing the psychometric function in real-time, i.e. during the test, and updating it after each trial has been completed to refine its best guess of where the threshold lies. We chose this method because of (1) its efficiency, e.g., Kontsevich and Tyler^83^ reported locating thresholds in fewer than 30 trials, (2) its flexibility (the ability to deal with acute sensitivity on the order of 10 cents for NH listeners to as much as 10 semitones for cochlear implant users (the end goal of this research —see discussion), and (3) its robustness to inattention and operational mistakes (which may become relevant when testing pediatric populations in the future). To this aim, we transformed the scale of semitones into a decibel scale (here, having nothing to do with sound intensity) ranging from − 20 dB (i.e., 1 cent) to + 20 dB (i.e., 100 semitones), with a 0.01 dB granularity. Our initial prior was set to be as uninformed as possible, with a mean of 10.8 dB (i.e., 12 semitones) and a standard deviation of 80 dB.

Four QUEST procedures, as described in Watson and Pelli^81^, were run in parallel on the basis of a Weibull function with gamma fixed at 50% (chance level), delta fixed at 1% (upper asymptote), and beta values (controlling the slope of the psychometric function) being 1, 2, 3, or 4, adjusting epsilon in each case so that threshold always corresponded to 70.7% correct performance point on the psychometric function. After each trial, the four QUEST functions were amended by the success or failure function respective to the ΔF0 value just tested, and the best guess was determined by picking the highest value among the four likelihood maxima (one in each QUEST function). Herein, the ΔF0s varied within much smaller ΔF0s (as low as 1 cent). In other words, this procedure tracked the most probable threshold and slope. In pilot testing, we found that this procedure was, to some degree, *too efficient* and also prone to being stuck in local minima. Therefore, instead of placing the next trial at the best guess, we injected a random roving of ± 5 dB (e.g., any value between 3.2 cents and 32 cents for a best guess at 10 cents; or any value between 32 cents and 3.2 semitones for a best guess at 1 semitone). This is helpful to participants by occasionally presenting a few easy trials to remind them of the cue they are supposed to attend to, instead of focusing exclusively on the region where the target is barely detectable.

The Bayesian staircase was run for a total of 210 trials and lasted about 12 min. Arguably, experimental time of 12 min may not appear strikingly short, thereby questioning the efficiency of this Bayesian procedure. In reality, we could have stopped the procedure earlier, but accuracy in our estimate of F0 JND was of critical importance here since it was the value eventually used to adjust the size of the feedback alteration in the third experiment, hence our cautious and conservative decision for 210 trials.

Before the test, participants completed practice blocks of 20 trials (10 trials with a positive ΔF0 and 10 trials with a negative one) in order to familiarize them with the 3I-2AFC paradigm and the type of stimuli. This time and only in the practice blocks, the ΔF0 was fixed at 4.8 semitones. Participants were instructed to select the interval that was different in pitch from the reference and give their responses by using a mouse. The test was only initiated, provided that participants obtained > 80% of correct responses, averaged over the two directions (up or down). During both training and test trials, visual feedback was provided to participants with a face animation showing happy or sad faces indicating correct or incorrect responses, respectively.

### Data Analysis of adaptation paradigm

All the recordings were imported to MATLAB 2018b. First, the recorded vocalizations over all trials were concatenated and passed through the PSOLA pitch detection algorithm of PRAAT to extract F0 points every 10 ms, within the default F0 search range of 75 to 600 Hz. This led to a histogram of F0 points pooled over time and all trials, which was fitted with a Gaussian distribution. The mean of this Gaussian F0 distribution defined the participant’s characteristic voice pitch. We considered it to be the middle of the participant’s vocal range, and as a result the window for F0 search was narrowed down to ± 6 semitones around the identified characteristic voice pitch and each recording was re-processed. This enabled to prevent octave errors while tracking the F0 contour per trial for the whole vocalization duration. Subsequent analyses focussed on two segments of the productions: the first 100 ms where compensatory changes could not have happened (as they do not occur before 100–150 ms, e.g. see Fig. 2 in Sares et al.^77^) and the middle 100 ms where compensatory changes could possibly be confounded with adaptation-related changes.

To assign responses in the hold phase or the washout phase to an opposing or following behavior, a traditional approach is to convert F0 values in cents based on a reference. This reference is often taken *as a single value* from the average of F0 values calculated over the baseline trials^42^. As mentioned in the introduction, it is not uncommon to see long-term upward or downward trends for F0 production across an entire experiment, making it difficult to assess in the end of the hold phase or over the washout phase whether a given behavior should be treated as opposing or following. To circumvent this problem, we developed a novel method based on a third-degree polynomial fit from the baseline trials and the last 50 trials of the washout phase. This approach enabled us to get an estimate of the reference per trial, rather than a single value, i.e. predicting what the F0s would have been throughout all the 270 trials, had the feedback always remained intact. Note that other parameters for the polynomial fit were attempted (see Supplementary Information 3). In a nutshell, the anchor used for the end of the washout phase had to be sufficiently long for the fit to be reasonable, otherwise, a linear or quadratic fit could not represent adequately the curvatures observed. Therefore, we set the cubic polynomial based on all baseline trials and the last 50 washout trials. Further, this enabled us to examine potential after-effect within the first 30 trials of the washout phase.

**Figure 9:**
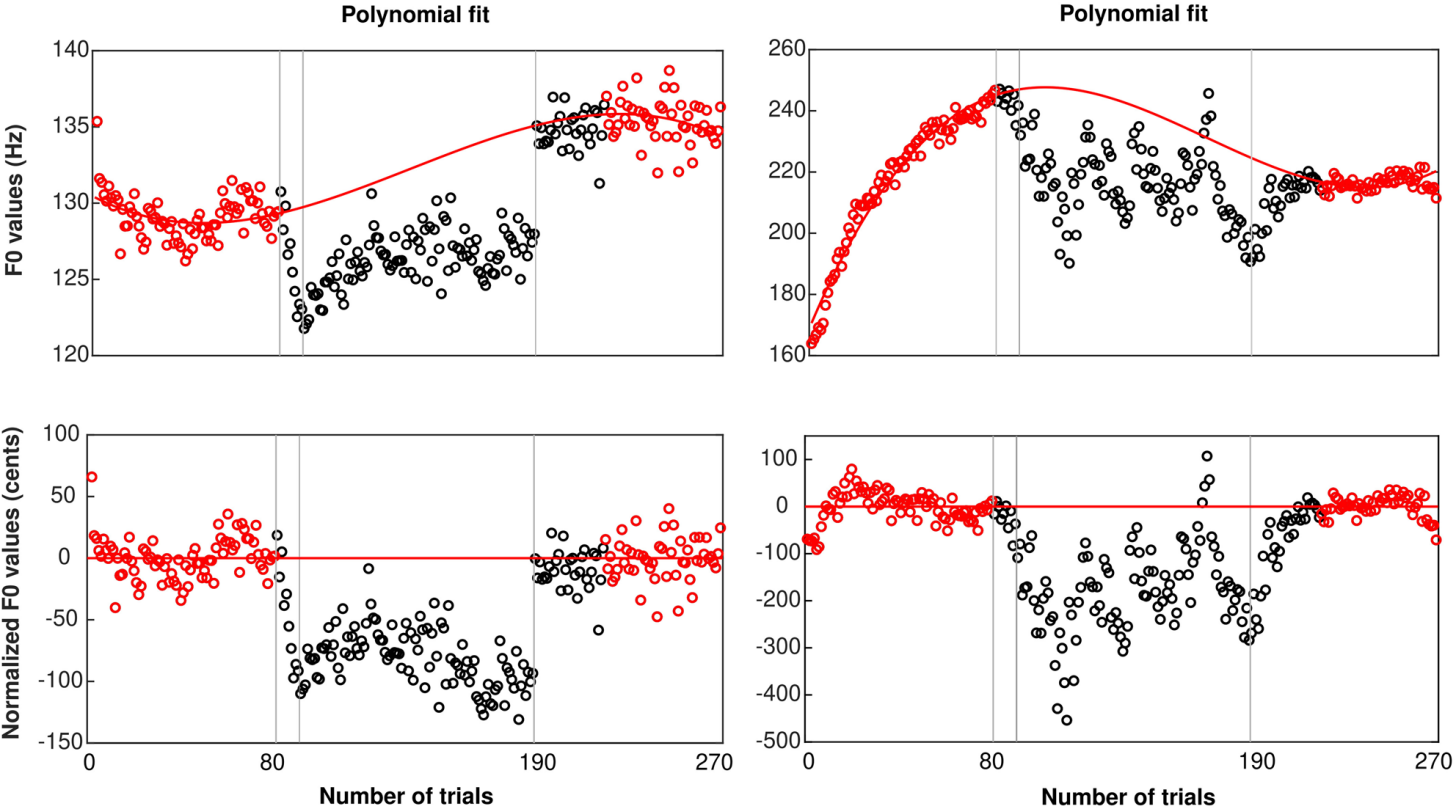
Samples of normalization method used in this study. The top panels represent F0 data in Hz and the bottom panels show the same data converted in cents relative to a reference based on a third-degree polynomial fit. Red lines represent what the production data would have been, had the feedback always remained intact. The left column shows the vocal behavior of a male speaker with stable production within baseline trials. The right column illustrates a male speaker with a wide vocal range.

Figure 9 depicts the normalization method applied on two different vocal behavior recorded from our participants. The left top panel shows the vocal behavior of a male speaker with stable production within the 80 baseline trials. In such a case, a simple average would suffice in generating a baseline F0 around 130 Hz upon which one could easily observe the opposing response (here to a + 100-cents shift) when a perturbation was applied. The right top panel, however, illustrates a male speaker with a wide vocal range, as he started his productions with an F0 around 165 Hz and reached an F0 around 245 Hz at the end of the baseline phase. For such unstable speakers, an average F0 calculated over the 80 trials of the baseline phase would fail to highlight the nature of the response, i.e., opposing or following. Our normalization procedure enabled a better estimation of the adaptation behavior when a perturbation was applied. As can be seen in the bottom panels, both cases (stable or unstable speaker) demonstrated here an opposing response of considerable magnitude even though across-trial variability remained an evident signature of the unstable speaker.

### Data analysis of F0 JND measurement

Data analysis of the perceptual task was done immediately after finishing the 3I-2AFC task, while the participant took a 3-min break to drink water. All the data were processed separately for positive and negative ΔF0s, using a finer range of delta values than during the Bayesian procedure (1, 2, 3, 4, and 5% lapse rate) and five beta values (with steps of 0.2, centered on the final beta estimate at the end of the staircase). Thus, 25 QUEST procedures were run at posteriori in order to refine the shape of the underlying psychometric function. Note that a similar range of QUEST parameters would have been difficult to run in parallel during data collection without impacting computational time (which itself was already lengthened by searching for a pseudo-stationary signal among the 80 recordings). A d’ fit was then reconstructed from the fit obtained with positive ΔF0s (hits) and 100% minus the fit obtained with negative ΔF0s (false alarms). The F0 JND, final threshold, was then extracted at a d’ of 0.77. Furthermore, this post-processing routine was run for each vowel in isolation in order to test whether sensitivity to one’s own voice pitch depended on the vowel produced (but it had no impact on the rest of the experimental protocol).

**Figure 10:**
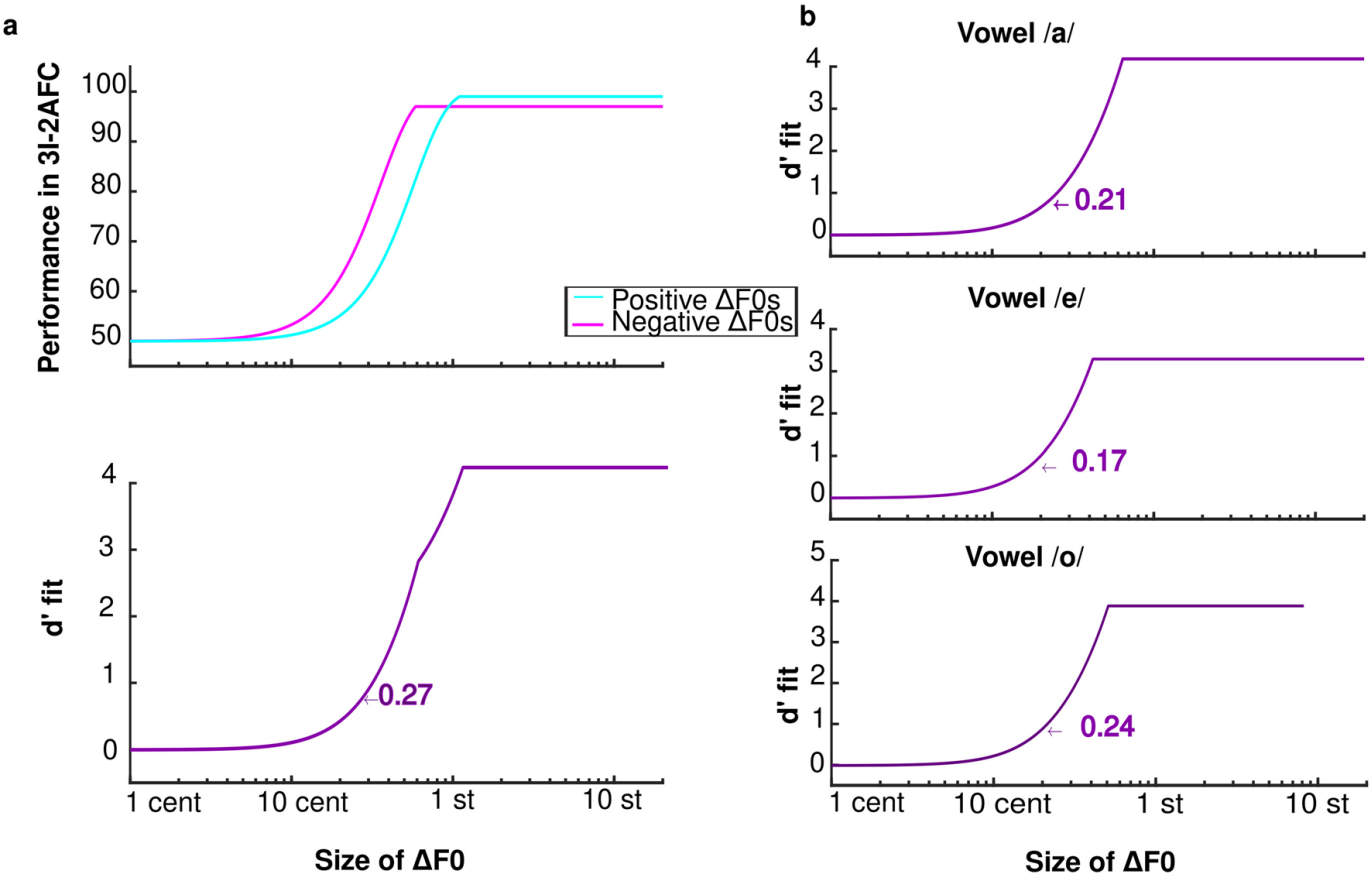
A sample result of the F0 sensitivity threshold of a 21-year old participant. (**a**) The top-left panel illustrates the shape of the Weibull fits obtained from the QUEST procedure. The combination of both resulted in a d’ fit shown on the bottom-left panel. (**b**) Panels show d’ fits derived for the three vowels.

The top-left panel of Fig. 10 shows an example of data from a participant illustrating the shape of the Weibull fits obtained from the QUEST procedure. The fit for positive ΔF0s had a delta of 3%, a beta of 4.2, and a 70.7% point corresponding to 27 cents. The fit for negative ΔF0s had a delta of 1%, a beta of 4.2, and a 70.7% point corresponding to 43 cents. The combination of both resulted in a d’ fit shown on the bottom-left panel: the poorer performance observed with negative ΔF0s is attributed to a sub-optimal choice of internal criterion^84^ and a d’ value of 0.77 is obtained at 27 cents. In other words, this 21-year-old woman could detect pitch errors in her own voice on the order of a quarter of a semitone. Moreover, the right panels show d’ fits derived for vowels /a/, /e/, and /o/, resulting in JNDs of 21, 17, and 24 cents, respectively. This result is considered as a good illustration of the efficiency of this Bayesian procedure since each fit was derived from only 70 trials on average (4 min of experimental time), and the vowel specific JNDs are reasonably close to global JND. Note that it was not uncommon to find all three vowel-specific JNDs smaller than the global JND (as in here). This presumably reflects that the bias in internal criterion is vowel specific. As a consequence, the global JND reflected that participants were to some degree confused by the constant alternation between vowels (across trials only), a confusion that hindered specifically the participants’ ability to stick to the same decision making strategy per vowel.

## Supplementary information

Supplementary information

Supplementary file2

Supplementary file3

Supplementary file4

## Data Availability

To protect the privacy of participants, anonymized data will be available to investigators upon request.
