# Supplementary material for: Adaptation to pitch-altered feedback is independent of one’s own voice pitch sensitivity
Source: Sci Rep. 2020 Oct 8;10:16860. doi: 10.1038/s41598-020-73932-1 (PMC7544828; doi:10.1038/s41598-020-73932-1)
Supplement: Supplementary file 1 — Supplementary information [file 41598_2020_73932_MOESM1_ESM.docx]

**Supplementary materials**

**Title:**

**Adaptation to pitch-altered feedback is independent of one’s own voice pitch sensitivity**

**Authors:**

**Razieh Alemi*^1,2,3^, Alexandre Lehmann^1,2,3^, and Mickael L. D. Deroche^1, 2, 3, 4^**

1. Department of Otolaryngology, Faculty of Medicine, McGill University, Montreal, QC, Canada
2. Centre for Research on Brain, Language & Music (CRBLM), Montreal, QC, Canada

3. International Laboratory for Brain, Music & Sound Research (BRAMS), Montreal, QC, Canada

4. Laboratory for Hearing and Cognition, Department of Psychology, Concordia University, Montreal, QC, Canada.

**^*^Corresponding author**: [razieh.alemi@mail.mcgill.ca](mailto:razieh.alemi@mail.mcgill.ca)

**Date of submission:**14/04/2020

**Date of 1^st^ re-submission:**17/07/2020

**Date of 2^nd^ re-submission:**14/09/2020

**Supplementary 1: Testing the null hypothesis**

In this study, there were a number of null results, which are notoriously difficult to interpret with traditional statistics. Thus, we reiterated specific tests with Bayesian statistics using JASP (<https://jasp-stats.org>). First, we looked at the consistent lack of after-effect (calculated from the first 30 trials of the washout phase with the early-F0 metric). A Bayesian one-sample t-test revealed a Bayes Factor (for the washout phase) of 0.150 and 0.144 for the first and middle segments, respectively, in the first experiment, and 0.201, and 0.376, for the first and middle segments, respectively, in the third experiment. These are considered *moderate evidence for the absence of after-effect*. Second, we looked at the F0 JNDs. A Bayesian repeated-measures ANOVA revealed a Bayes Factor was 0.067 for including vowel as a main effect, and 0.028 for including vowel by group as an interaction, providing *strong evidence that vowel did not play any role* in the present F0 discrimination task. In contrast, the Bayes Factor was 0.361 for including group as a main effect, providing an *absence of evidence* for a group effect. Note that a Bayesian correlation did not support an effect of age in these data (Bayes Factor = 1.332). Third, at the heart of this study was the lack of relationship between F0 JNDs and the adaptation behavior (illustrated in Fig. 5 of the main text). The Bayes Factors were 0.208 with adaptation magnitudes from the first experiment and 0.228 with adaptation magnitudes from the third experiment. Once again, these factors are considered *moderate evidence that adaptation is independent of F0 sensitivity.*

**Supplementary 2: Intensity of the vocal output**

An additional analysis was performed to explore possible reasons why adaptation happened to be stronger (at least in the first experiment) with vowel /a/ than vowel /e/ or /o/. We speculated that the control of vowel /a/ might be facilitated by specific positions of articulators and a large opening of the mouth. Thus, we wondered whether important information could be gleaned from the intensity values of the recordings for each vowel, each group, each phase (baseline versus hold phase), and each experiment. Thus, we ran a mixed ANOVA with three within-subject factors (experiment, phase, and vowel) and one between-subjects factor (group). Mauchly’s test of sphericity was not significant for the effect of vowel [Chi2(2) = 1.2, p = 0.544] but significant for the interaction between vowel and phase [Chi2(2) = 7.4, p = 0.025]. There was a main effect of vowel type [F(2,114) = 64.0, p<0.001], reflecting that vowel /o/ was always the most intense vowel (68.7 dB, on average across the two phases and the two experiments), followed by vowel /a/ (67.4 dB), and /e/ was the weakest (66.5 dB). Given that adaptation magnitude was similar for /o/ and /e/ while these two vowels differed the most in intensity, this would suggest that intensity played no direct role into the adaptation phenomenon.

However, we also found that vowel interacted with phase [F(1.8,101.5) = 9.2, p<0.001]: there was no change in intensity for vowel /o/ [F(1,57) = 0.7, p = 0.410] going from 68.6 dB at baseline to 68.8 dB during the hold phase, and vowel /e/ [F(1,57) = 1.9, p = 0.174] going from 66.4 dB at baseline to 66.7 dB during the hold phase; however, vowel /a/ was more intense during hold phase trials than during baseline trials [F(1,57) = 7.0, p = 0.010] going from 67.0 to 67.7 dB, reflecting that intensity was weaker over the baseline, and this was especially true for the early trials of the baseline – see Supplementary Fig. S1]. Finally, any other main effect or interaction (2-, 3-, or 4-way) missed significance (p>0.102). The vowel-phase interaction is certainly intriguing considering that vowel /a/ is the vowel that led to the greatest adaptation magnitude. In consequence, we cannot exclude that intensity played a role in the adaptation phenomenon, but it is not an explanation that works for differences between vowels but rather the same constant vowel across baseline and hold phase.

**Supplementary 3: Normalization of F0 data**

The F0 data described in this article were expressed in cents relative to a baseline. Traditionally, this baseline is taken as a single value from the average of baseline trials. This is highly problematic in speakers who do not exhibit a stable voice pitch during baseline trials, such as the speaker illustrated in the right panel of Figure 9 in the main text. Moreover, speakers may have a stable baseline and yet exhibit a trend (upwards for some speakers, downwards for others) throughout the whole experiment, such that the voice pitch produced towards the end of the study (e.g. once the feedback is back to normal) can differ massively from that produced at the start of the experiment. In this project, we developed a novel method to measure the adaptation response more accurately by fitting a cubic polynomial based on the baseline trials combined with the last 50 washout trials. Here, we justify our choice by exploring the impact of other parameter choices.

Supplementary Figure S2 illustrates the data from the same subject (used as an example in the right panel of Fig.9, in the main text) processed in different ways: the top panels show fits based on the baseline + last 10 washout trials, while the bottom panels show fits based on the baseline + all 80 washout trials. First, a linear fit simply does not do justice to subjects who fluctuate considerably during the baseline phase. A quadratic fit can in principle represent the curvature that happens over the baseline phase but it would largely overshoot over the perturbed trials, especially if only a few trials serve as anchor for the end of the washout phase (top-middle-left). If all washout trials are used, the quadratic curve may become a poor fit to both baseline and washout phases. This example, therefore, demonstrates that neither a first- nor a second-degree polynomial are sufficient to model how the data could have been like, had there been no F0 perturbation. Now, the cubic and quartic polynomials generally do a decent job at fitting the baseline trials. However, they tend to generate undesirable curvatures over the washout phase when only 10 trials are used as anchor (top middle-right and top-right panels). This does not occur when enough trials are considered: typically, when using the last 80 trials, the fit is decent over the washout phase, but can sometimes be too stringent (bottom-right panel). Furthermore, given that there may be (at least in principle) some carry-over effect of the F0 perturbation, it is important to exclude the first few blocks of the washout phase, at the very least to not hinder our capability to observe adaptation over the perturbed trials. This is why we finally decided to opt for a cubic fit based on all baseline trials but only the last 50 trials of the washout phase. Note that a quartic (or possibly higher-degree) polynomial would work as well but we strived for parsimony; therefore, we chose the cubic one.

To further support this explanation based on a single participant’s data, we investigated what impact our choice of parameters could have had on the whole dataset (see Supplementary Fig. S3) and its statistical results using the same ANOVA described in the article namely two within-subject factors (trial window and vowel), and one between-subject factor (group). For this exploration, we limited ourselves to the data of the first experiment.

**1- 1^st^ degree polynomial fit:**

Fit based on baseline trials + last 10 trials: Results showed no main effect of group [p = 0.736], a main effect of vowels [p<0.001] but no effect of phase [p = 0.454], and any 2-way interaction was not significant [p>0.681], but the 3-way interaction was [p = 0.014].

Fit based on baseline trials + last 80 trials: Results showed no main effect of group [p = 0.552], a main effect of vowels [p<0.001] but no effect of phase [p = 0.282], and any 2-way interaction was not significant [p>0.482], but the 3-way interaction was [p = 0.030].

**2- 2^nd^ degree polynomial fit:**

Fit based on baseline trials + last 10 trials: Results showed no main effect of group [p = 0.454], a main effect of vowels [p<0.001] but no effect of phase [p = 0.445], and any 2-way interaction was not significant [p>0. 429], but the 3-way interaction was [p = 0.018].

Fit based on baseline trials + last 80 trials: Results showed no main effect of group [p = 0.747], a main effect of vowels [p = 0.001] but no effect of phase [p = 0.257], and any 2-way interaction was not significant [p>0.098], but the 3-way interaction was [p = 0.035].

**3- 3^rd^ degree polynomial fit:**

Fit based on baseline trials + last 10 trials: Results showed no main effect of group [p = 0.781], no main effect of vowels [p = 0.188] or phase [p = 0.609], and any 2- or 3-way interactions were not significant [p>0. 410].

Fit based on baseline trials + last 80 trials: Results showed no main effect of group [p = 0.780], a main effect of vowels [p = 0.003] and phase [p = 0.029], and any 2- or 3-way interactions were not significant [p>0.097].

**4- 4^th^ degree polynomial fit:**

Fit based on baseline trials + last 10 trials: Results showed no main effect of group [p = 0.879], no main effect of vowels [p = 0.436] or phase [p = 0.294], and any 2- or 3-way interactions were not significant [p>0.114].

Fit based on baseline trials + last 80 trials: Results showed no main effect of group [p = 0.803], a main effect of vowels [p = 0.039] and phase [p = 0.029], and any 2- or 3-way interactions were not significant [p>0.102].

To summarize, the direction of the shift was always irrelevant. In contrast, the effect of vowel and that of trial window depended on the parameters considered. In line with the limitations described above for the 1^st^ and 2^nd^ degree polynomials, we found that the effect of trial window could be lost and that of vowels restricted to some combinations of group and trial window (which were indeed mistakes). Similarly, the choice of using only the last 10 trials for the washout phase generated some noise and both the effect of vowels and that of trial window were lost. In contrast, the results obtained with the cubic or quartic polynomials using all 80 washout trials resulted in qualitatively similar conclusions as those reported in the article.

**Figure Legends**

Supplementary Figure S1: Left pannels depict mean (± SE) of vocal intensity of all vowels produced by participants across the four phases of the first (a.) and third (b.) experiments, trials with intact auditory feedback are shown by gray rectangle. Right pannels show vocal intensity of the three different vowels produced in the baseline and hold phase of the first (a.) and third (b.) experiments. SE: standard error.

Supplementary Figure S2: Samples of polynomial fits based on baseline trials and an end anchor using either the last 10 trials (top panels) or all washout trials (bottom panels). Note the different degrees of the polynomial curve.

Supplementary Figure S3: Group-level average of the vocal performance of participants who experienced ±100 cents F0 shifts as assed by different degrees of the polynomial fit (as shown in columns) and different numbers of washout trials used as end anchor (as shown in rows). The red and blue lines (± SE) represents the vocal behavior in the downward and upward shift directions, respectively. SE: standard error.
