# Supplementary figures and images for: Adaptation to pitch-altered feedback is independent of one’s own voice pitch sensitivity
Source: Sci Rep. 2020 Oct 8;10:16860. doi: 10.1038/s41598-020-73932-1 (PMC7544828; doi:10.1038/s41598-020-73932-1)

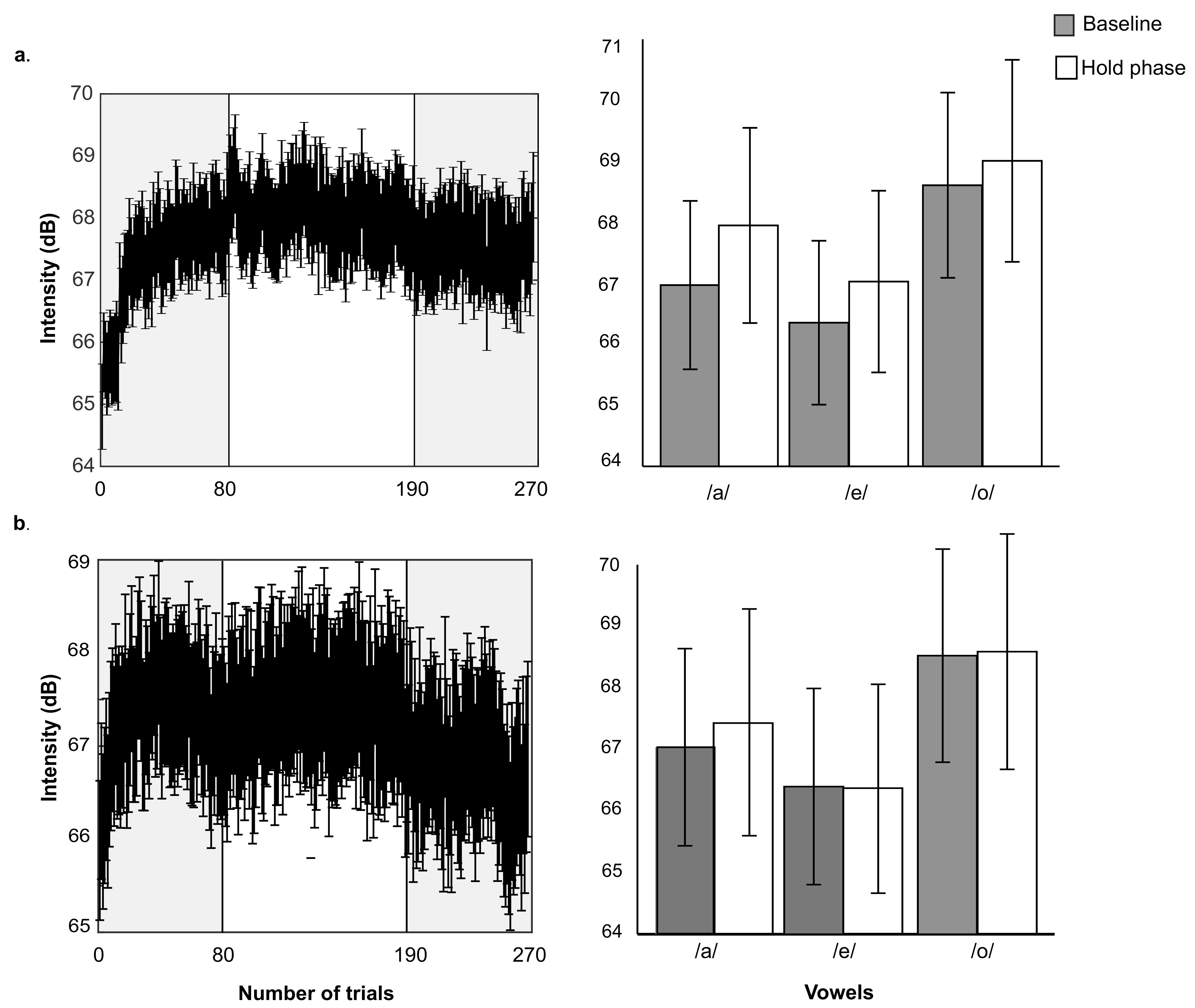

Supplement: Supplementary file 2 — Supplementary file2 [file 41598_2020_73932_MOESM2_ESM.jpg]

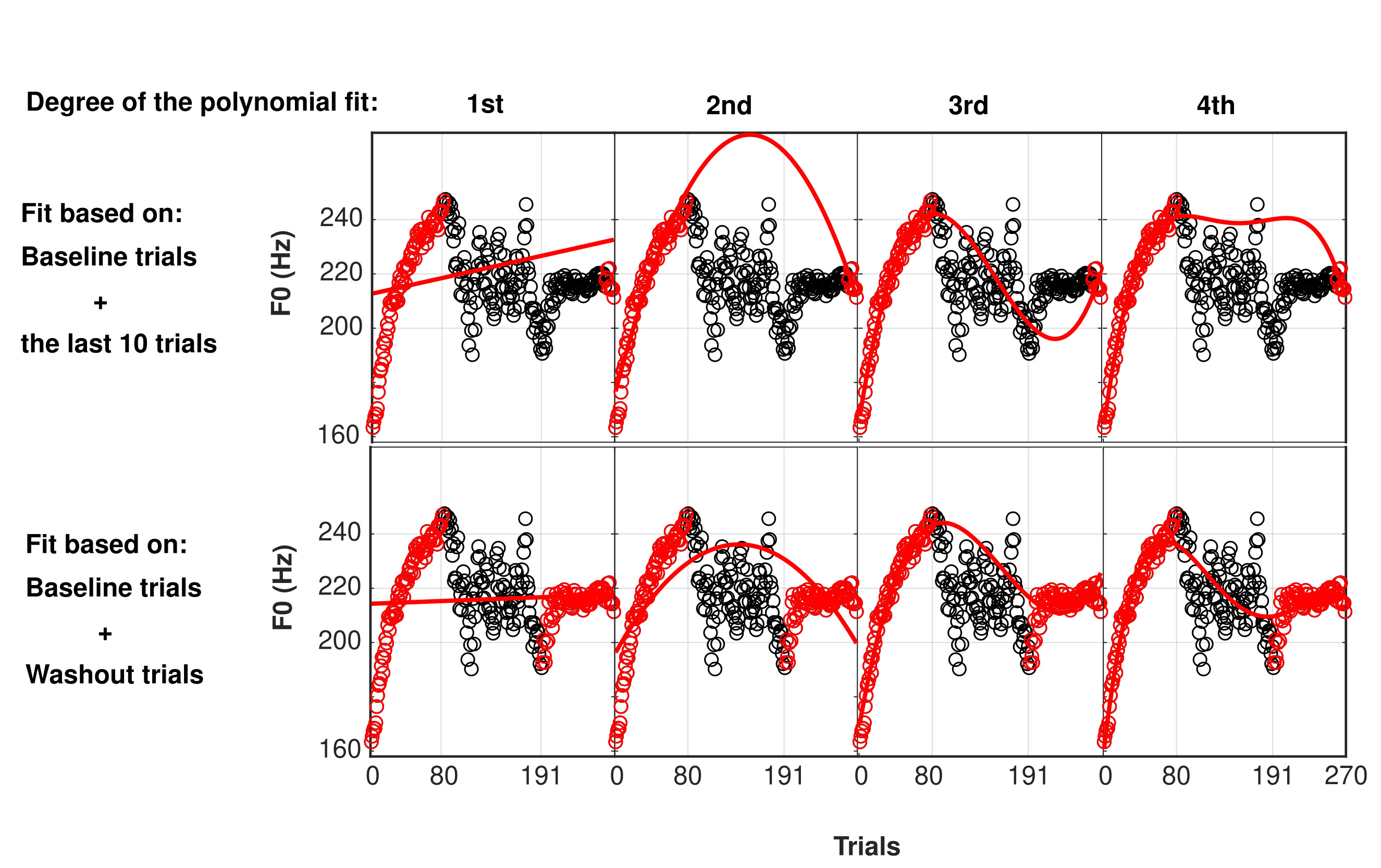

Supplement: Supplementary file 3 — Supplementary file3 [file 41598_2020_73932_MOESM3_ESM.jpg]

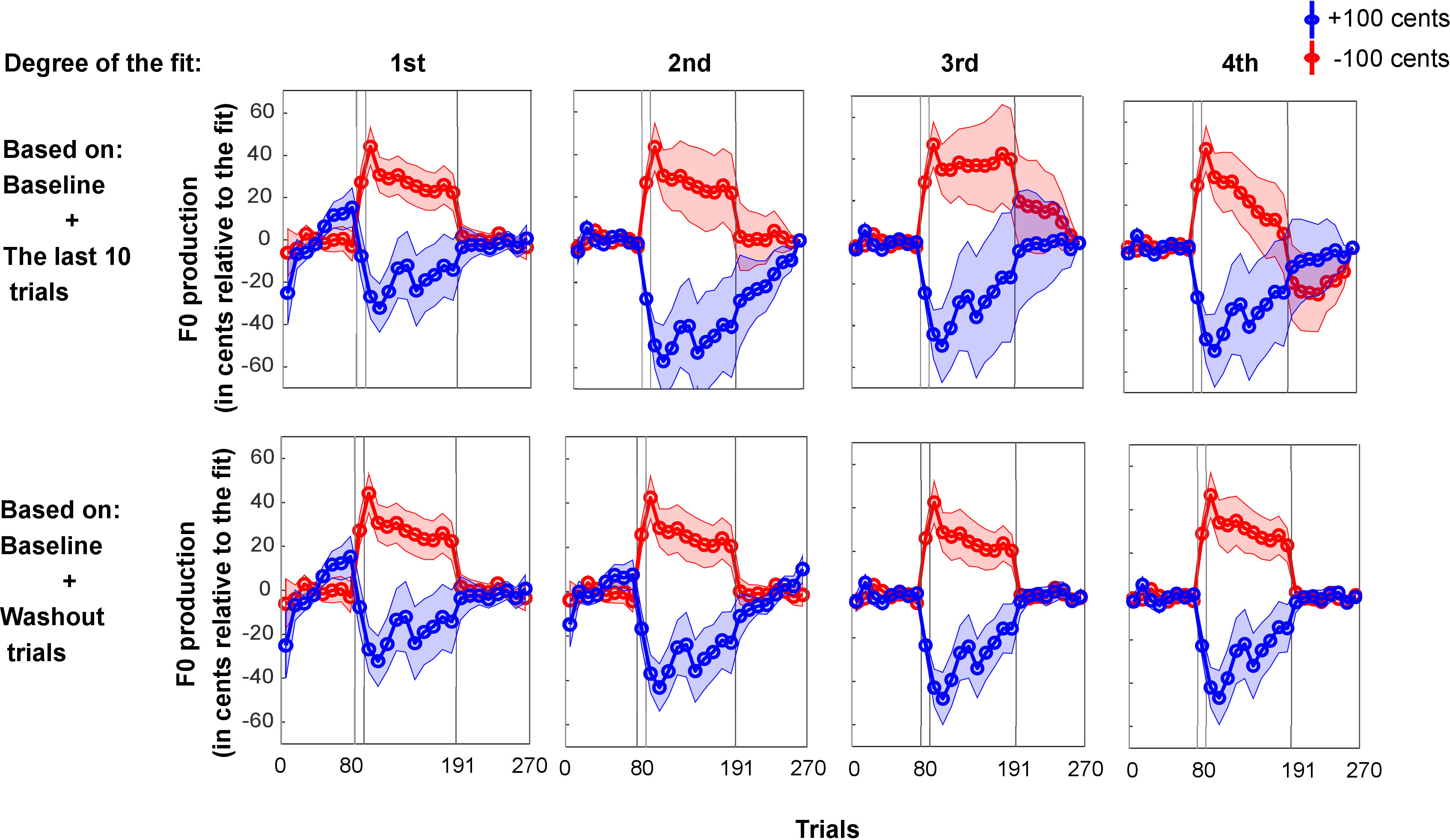

Supplement: Supplementary file 4 — Supplementary file4 [file 41598_2020_73932_MOESM4_ESM.jpg]
